# Aging as a Loss of Goal‐Directedness: An Evolutionary Simulation and Analysis Unifying Regeneration with Anatomical Rejuvenation

**DOI:** 10.1002/advs.202509872

**Published:** 2025-10-13

**Authors:** Léo Pio‐Lopez, Benedikt Hartl, Michael Levin

**Affiliations:** ^1^ Allen Discovery Center at Tufts University Medford, MA 02155 USA; ^2^ Institute for Theoretical Physics TU Wien Vienna 1040 Austria; ^3^ Wyss Institute for Biologically Inspired Engineering at Harvard University Boston, MA 02115 USA

**Keywords:** aging, cognition, computational modeling, in silico, morphogenesis, neural cellular automata (NCA)

## Abstract

Although substantial advancements are made in manipulating lifespan in model organisms, the fundamental mechanisms driving aging remain elusive. No comprehensive computational platform is capable of making predictions on aging in multicellular systems. Focus is placed on the processes that build and maintain complex target morphologies, and develop an insilico model of multiscale homeostatic morphogenesis using Neural Cellular Automata (NCAs) trained by neuroevolution. In the context of this model: 1) Aging emerges after developmental goals are completed, even without noise or programmed degeneration; 2) Cellular misdifferentiation, reduced competency, communication failures, and genetic damage all accelerate aging but are not its primary cause; 3) Aging correlates with increased active information storage and transfer entropy, while spatial entropy distinguishes two dynamics, structural loss and morphological noise accumulation; 4) Despite organ loss, spatial information persists in tissue, implementing a memory of lost structures, which can be reactivated for organ restoration through targeted regenerative information; and 5) rejuvenation is found to be most efficient when regenerative information includes differential patterns of affected cells and their neighboring tissue, highlighting strategies for rejuvenation. This model suggests a novel perspective on aging as loss of goal‐directedness, with potentially significant implications for longevity research and regenerative medicine.

## Graphical abstract

The paper proposes that the root cause of aging is the loss of anatomical goal‐directedness after development. Using evolutionary neural cellular automata simulations, the authors show that after the organism has reached its developmental homeostatic setpoint (the adult morphology), the absence of target state to pursue leads to a drifting anatomical decline. This is reversible by re‐imposing a regenerative setpoint via the injection of embryonic state information.

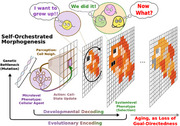

## 1 Introduction

### 1.1 Theories of Aging

**Figure 1:**
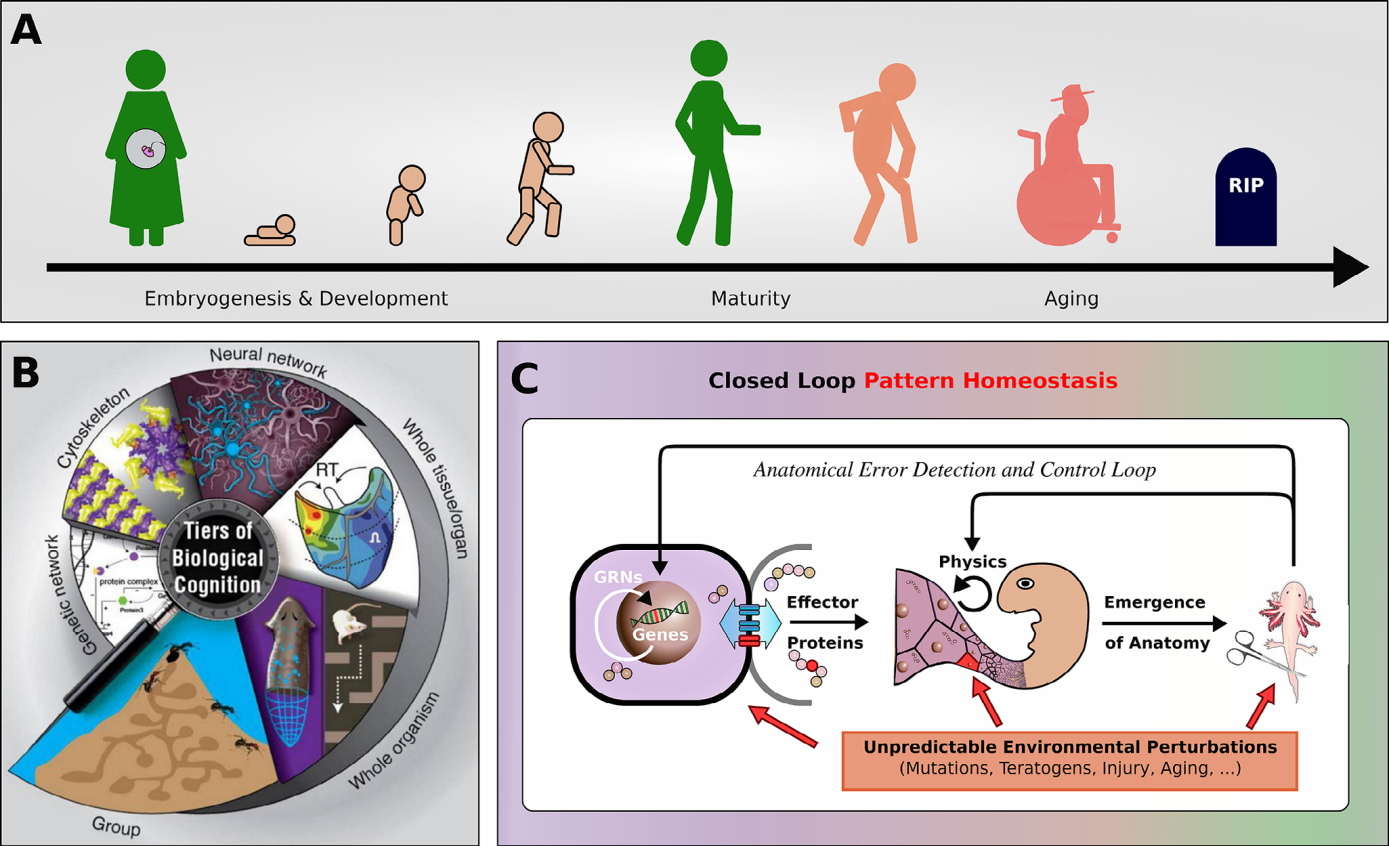
A) A schematic illustration of the stages of life of a complex organism, starting from embryogenesis, development and growth, through maturity, decline, aging, and eventually death. B) Multicellular organisms are composites of hierarchically interlocked layers of biological self‐organization, spanning physical scales from the molecular level, over cells, tissues, organs, to organisms and even groups or collectives of individuals. Image by Jeremy Guay of Peregrine Creative. Used by permission from.^[^ ^20^ ^]^ C) To develop an organism's morphology and sustain its integrity against environmental perturbations, biology maintains a multi‐scale closed loop pattern homeostasis mechanism, where the components at every scale of self‐organization are capable of self‐regulatory and error‐correcting behavior. Used by permission from.^[^ ^21^ ^]^ Fundamentally, cells themselves have numerous behavioral and information processing capabilities: To control multicellular morphology during embryogenesis, as well as in mature and even aging tissue, cells utilize a variety of communication channels, such as bioelectrical, biochemical, and biomechanical processes, enacting local communication protocols via intercellular signaling, intracellular information processing, and cell‐state regulation .^[^ ^22^ ^]^ This can be seen as a process of physiological computation by which cells interact and respond to their environment to create complex patterns and structures, guiding embryogenesis, maturation, metamorphosis, remodeling, regeneration, and suppression of cancer and aging .^[^ ^23^ ^]^ In most organisms, with only very few exceptions, these homeostatic processes eventually break down, leading to irreversible morphological decline, aging, and death.

Aging is a system‐level, near‐ubiquitous biological process characterized by morphological and functional alterations in cellular and extracellular components, resulting in a systematic decline in biological functions.^[^ ^1^ ^]^ This degenerative process is modulated by genetics, lifestyle, and environmental factors.^[^ ^2^ ^]^ As cellular functionality diminishes with age, there is a corresponding decrease in physiological capacities and an enhanced vulnerability to a spectrum of diseases, including cardiovascular disorders, cancer, neurodegenerative diseases, type II diabetes, and various infections. These conditions collectively compromise the quality of human life.^[^ ^2^, ^3^ ^]^ **Figure** **1** A schematically illustrates the typical life cycle of a complex organism comprising various stages from embryogenesis through maturity, decline, aging, and eventual death.

Several theories have been proposed to explain the aging process, which we can classify into damage‐based and programmatic theories,^[^ ^1^, ^4^, ^5^ ^]^ with the former being more extensively studied.^[^ ^1^, ^3^, ^6^, ^7^ ^]^ Damage‐based theories assert that aging results from the accumulation of molecular damage, impacting crucial cellular components like the genome, telomeres, mitochondria, and proteins, driven by imperfect repair mechanisms.^[^ ^6^, ^7^, ^8^, ^9^ ^]^ Conversely, programmatic theories suggest that aging is genetically predetermined, or quasi‐programmed and encoded within the genome, not merely due to stochastic accumulation of damage.^[^ ^10^, ^11^, ^12^, ^13^ ^]^ Here, we formulate and computationally simulate a fundamentally different hypothesis based on the nature of morphogenesis as a homeodynamic system working toward anatomical setpoints: that aging is the result of a proto‐cognitive dysfunction among the cellular collective, and does not require damage or evolved antagonistic mechanisms in order to occur. Instead, we propose that, as observed in animal behavior,^[^ ^14^ ^]^ cellular living systems without goals to pursue tend to degrade. Our hypothesis is that aging is the result of intrinsic dynamics of complex cybernetic (goal‐directed) systems, and is not caused by external noise or damage. Nevertheless, it is consistent with other theories of aging^[^ ^15^, ^16^ ^]^ which propose additional dynamics that can lead to loss of setpoints of anatomical homeostasis, and can be accelerated by various kinds of damage.

Antagonistic pleiotropy posits that certain genes have beneficial effects in early life but detrimental effects in later life. These genes confer advantages such as increased fertility and resilience in youth, which are critical for survival and reproduction. However, as an individual ages, the same genes contribute to aging and decline because their negative effects accumulate. The theory suggests that natural selection favors these genes because of their early‐life benefits, despite their harmful subsequent impacts. This results in a trade‐off between early benefits and later costs.^[^ ^17^ ^]^ Another related idea is the hyperfunction theory, which suggests that aging is not primarily a result of accumulating molecular damage or genetic programming, but rather is due to a continuous and excessive operation of cellular growth processes that were beneficial during the earlier stages of life.^[^ ^15^, ^16^ ^]^ Central to this theory is the role of the Target of Rapamycin (TOR) pathway, which is a major regulator of growth and metabolism in cells. During the growth phase of an organisms life, the TOR pathway promotes cellular and organismal growth by driving processes like protein synthesis, nutrient uptake, and cell division. These functions are vital for development and reproductive success. However, aging arises when these growth‐promoting activities are not appropriately down‐regulated after the organism has passed its reproductive prime. Instead of adjusting to a maintenance mode that would be more suitable for the non‐reproductive phase of life, the TOR pathway continues to stimulate cellular processes actively. This ongoing activity can lead to “hyperfunction”, where cellular components and systems are overstimulated.^[^ ^15^, ^16^ ^]^ It is not clear, in that scenario, why the efficient processes of morphological homeostasis^[^ ^18^, ^19^ ^]^ do not orchestrate these active processes toward upkeep of functional anatomy (c.f., Figure 1B,C).

Our approach is consistent with the antagonistic pleiotropy and hyperfunction models in that we also propose that fundamental morphogenetic processes are involved in aging. However, we do not focus on specific genes with early versus late roles, but on the role that large‐scale morphogenetic plasticity plays in establishing and maintaining bodies and the possible disorders of such cybernetic (goal‐driven) processes. We and others have emphasized the indirect link^[^ ^21^, ^23^, ^24^, ^25^ ^]^ between genotype and phenotype: not just complexity and pleiotropy, but the fact that the morphogenetic layer has active homeodynamic capabilities that enable collections of cells to be aligned with respect to setpoints in the space of anatomical possibilities^[^ ^18^, ^26^, ^27^ ^]^ (c.f., Figure 1B,C). Living materials must navigate the space of possible organ and tissue arrangements as needed, starting from diverse initial states single cells (egg‐based development), a different body (in the case of metamorphosis), or a damaged body (in the case of regeneration). In all of these cases, drastic growth and remodeling must occur while failing or no longer needed sub‐components must be replaced on‐the‐fly. This process has been described as a collective intelligence,^[^ ^28^, ^29^ ^]^ because of its problem‐solving capacities; like other advanced intelligences, it will be subject to specific failure modes whose origin is not organic disease or damage, but the informational dead end of completing ones mission and not having a new setpoint toward which to strive.

We formulate, and simulate the consequences of, the hypothesis that the aging phenotype results from morphogenetic processes ceasing their targeted navigation of anatomical morphospace, after development is completed. But adulthood, and the rigors of daily life, are precisely when these processes are needed, in the context of stress, degeneration, and cell replacement, to maintain the Ship of Theseus that is the body. What will bind the cellular components of a body toward common paths in anatomical space after their goals have been reached and the body built? Tissue renewal will continue locally, for a time, but the thing that bound individual cells into a coherent embryo a commitment to a specific navigation path in anatomical space disappears at the global scale. Damage‐based theories of aging are thus hypothesized to be secondary: they induce aging to the extent that they rob cells of clear and consistent anatomical and histological setpoints for which to aim. But the primary new proposal here is that complex goal‐driven systems can experience a kind of intrinsic disorganization after they have met their goal. In the absence of strong effort toward targets in morphospace, the cellular collective begins to disband, giving way to systemic disease states such as degeneration and cancer the inevitable outcome of a body that increasingly becomes a group of individual cells, not a unified higher‐order morphogenetic agent.^[^ ^30^ ^]^

### 1.2 Knowledge Gaps

Recent research suggests a shift in our understanding of aging, proposing that it results from changes in biological information processing rather than solely from cellular and molecular damage. This concept, linked to a malfunction in the “software of life”, focuses on how aging processes are governed by the epigenetic changes, either bioelectrical or chromatin modifications.^[^ ^5^, ^31^, ^32^ ^]^ The differing dynamics, capabilities, and failure modes of the molecular layer and the physiological computational layers have long been studied in neuroscience, but are only now beginning to be related to areas such as developmental biology and cancer.^[^ ^33^, ^34^, ^35^ ^]^ Novel computational frameworks are needed to begin to integrate these ideas in aging and longevity research. Although substantial advancements have been made in extending lifespan in simple model organisms through genetic, dietary, and pharmacological means,^[^ ^2^, ^36^, ^37^ ^]^ the fundamental mechanisms driving aging in humans remain elusive and widely debated. Current life extension strategies show limited effectiveness.^[^ ^38^ ^]^ Much of the research has adopted a bottom‐up approach, focusing on the biological hardware, the cellular components such as genes, molecular pathways, and, more recently, the epigenome. However, managing aging at such a micro‐level may not be practical for complex structures like human anatomy.^[^ ^19^, ^39^, ^40^ ^]^ This suggests that a broader, possibly more integrated approach may be necessary in order to fully understand and effectively intervene in the aging process.

Computational modeling has proven useful in the past to help characterize dynamics guiding complex morphogenesis.^[^ ^41^, ^42^, ^43^, ^44^, ^45^, ^46^, ^47^, ^48^, ^49^ ^]^ However, to the best of our knowledge, there is currently no computational model in the literature explicating the aging process as a loss of goal‐directedness or directionality within the morphogenetic control system. The field is mostly dominated by experimental approaches and focused on the mathematical and computational modelling of a specific hallmark of aging,^[^ ^6^ ^]^ network approaches to aging, or the impact of the environment on aging.^[^ ^50^, ^51^, ^52^, ^53^ ^]^ One prior study utilized (similar to our model) an NCA to perform morphogenesis (see next section): with gradient‐based deep learning, the self‐regulatory interaction dynamics of distributed cellular agents have been optimized to grow a target morphology in a fully self‐orchestrated way. However, without explicitly stabilizing morphogenesis by additionally training the cellular agents on regenerative tasks, anatomies kept growing uncontrollably after development. Thus, the study focused on an adversarial approach to stabilize self‐orchestrated morphogenesis rather than studying aging and its causes in such a multi‐cellular tissue per se.^[^ ^54^, ^55^ ^]^

A key knowledge gap lies in the absence of models that explicitly simulate the evolutionary pressures shaping developmental processes while accounting for the lack of anatomical morphostasis post‐development. Existing frameworks often overlook how multi‐scale competencies – spanning molecular, cellular, and tissue levels – transition from goal‐directed morphogenesis to maintenance, leading to intrinsic disorganization and degradation without clear setpoints. This oversight limits our ability to predict aging trajectories in complex multicellular systems and to explore interventions that reactivate latent regenerative potentials.

Existing mathematical models have not yet captured the biological signaling dynamics in aging tissue or revealed the underlying information dynamics that drive aging and the progressive degradation of anatomy over time. Unlike previous approaches, we uniquely address this gap by developing an evolutionary framework that integrates both molecular and systemic aspects of aging within a multi‐competency architecture. Our model demonstrates how cells, trained solely through evolution to execute developmental tasks, inherently lack a regenerative goal post‐development. By simulating this dynamic, we reveal that aging arises from the absence of targeted navigation in anatomical morphospace after development, offering a novel perspective on the root cause of aging. Moreover, our approach uncovers the potential for dormant regenerative programs to be reactivated, enabling targeted restoration of anatomical structures (c.f., Figure 1).

### 1.3 Biological Architecture of the Model

Morphogenesis is the result of multicellular collectives acting together to achieve a specific endpoint within the space of anatomical possibilities^[^ ^23^ ^]^ (see Figure 1). This process is driven by intercellular communication and information processing, where cells use different communication channels (biochemical, biomechanical or bioelectrical) to exchange information with their neighbors in a tissue^[^ ^22^, ^26^ ^]^ and implement a process of anatomical homeostasis where deviations from the species‐specific target morphology are progressively minimized by active changes in growth and form. This enables cells to adaptively respond in a context‐sensitive manner and regulate their behavior through local signaling protocols while collectively following a system‐level agenda to grow and maintain an organism's species‐specific anatomical form in a self‐orchestrated way. Thus, the development of embryonic tissues, the growth and remodeling of adult organs, and even the suppression of cancer and aging can be understood as morphological computation.^[^ ^22^ ^]^ In this sense, morphogenesis can be seen as an intermediate layer of physiological computation between genetic variation and a mature anatomical phenotype (see Figure 1C, significantly effecting the process of evolution.^[^ ^23^, ^46^ ^]^

Another important aspect of biology, so far neglected in the models of aging is multiscale competency^[^ ^23^, ^28^, ^56^ ^]^ (see Figure 1B,C). Biological systems implement an architecture in which context‐sensitive problem‐solving operates across various levels, from molecular networks to individual cells, tissues, organs, organisms, and even collective groups such as swarms. This structure allows biological entities to solve problems within specific domains and problem spaces relevant to each scale (metabolic, physiological, anatomical, and behavioral state spaces), contributing to the overall adaptability and resilience of life.^[^ ^22^, ^26^, ^57^ ^]^ At the molecular and cellular level, biological units navigate metabolic spaces to remain within adaptive regions despite changing circumstances. At higher organizational levels, these units form tissues and organs, which tackle more complex and larger‐scale physiological and morphological problems. For instance, cells can make autonomous decisions to maintain local balance while contributing to larger‐scale biological processes like tissue repair or development, and to how higher levels of organization constrain and facilitate the behavior of their parts.^[^ ^23^, ^58^, ^59^, ^60^, ^61^, ^62^, ^63^ ^]^ This enables organisms to adapt to changing conditions and maintain functionality even when faced with disruptions, as observed in regulative development and regeneration where cellular activity is managed toward specific anatomical outcomes despite injury, changes of cell number and size, etc.^[^ ^27^ ^]^ This is critical because resistance to both cancer and aging must involve both the upkeep of complex structures and a resistance to their progressive decline within the body.^[^ ^64^ ^]^

A key advantage of this multiscale architecture is that each level is capable of addressing its own problem‐spaces without needing constant guidance from higher organizational structures (local homeodynamic competency, see Figure 1C). This decentralized problem‐solving approach ensures that different biological processes, from cell division to behavioral adaptation, occur simultaneously and efficiently. Furthermore, this structure allows biological systems to be both robust and flexible, as failures at one level can often be compensated for by others. Additionally, the interplay between levels within this architecture enables the emergence of complex properties that cannot be explained by examining components in isolation. For example, the collective behavior of cells during processes like regeneration or morphogenesis illustrates how local interactions scale up to contribute to the organism's overall form and function.^[^ ^28^ ^]^ Similarly, defects of collective behavior can lead to developmental defects or aging. These interactions are not merely a sum of their parts; rather, they represent emergent capabilities that arise from the coordinated effort of biological subunits. The relative autonomy of layers in biology must surely impact on evolutionary and physiological constraints and drivers of aging, making it essential to study their properties with respect to the lifespan of individuals and evolutionary lineages.

This hierarchical but integrated organization is essential for the evolution of complex life forms. It promotes adaptability, allowing organisms to respond to environmental changes, evolve new features, and develop intricate systems that support survival and reproduction. Moreover, this multiscale competency architecture provides a framework for innovation in fields such as synthetic biology and regenerative medicine,^[^ ^29^, ^39^, ^65^ ^]^ where understanding and harnessing these principles could lead to breakthroughs in tissue engineering, organismal repair and aging.^[^ ^29^, ^31^ ^]^ This aspect of living forms – the critical layer of computational, problem‐solving competency that lies between the genotype and the phenotype – is an essential component of any theory of aging that integrates evolutionary and physiological dynamics to understand this complex phenomenon. Thus, we focused our computational model on the role of multiscale morphogenetic competency in the aging process.

### 1.4 Our Computational Model

**Figure 2:**
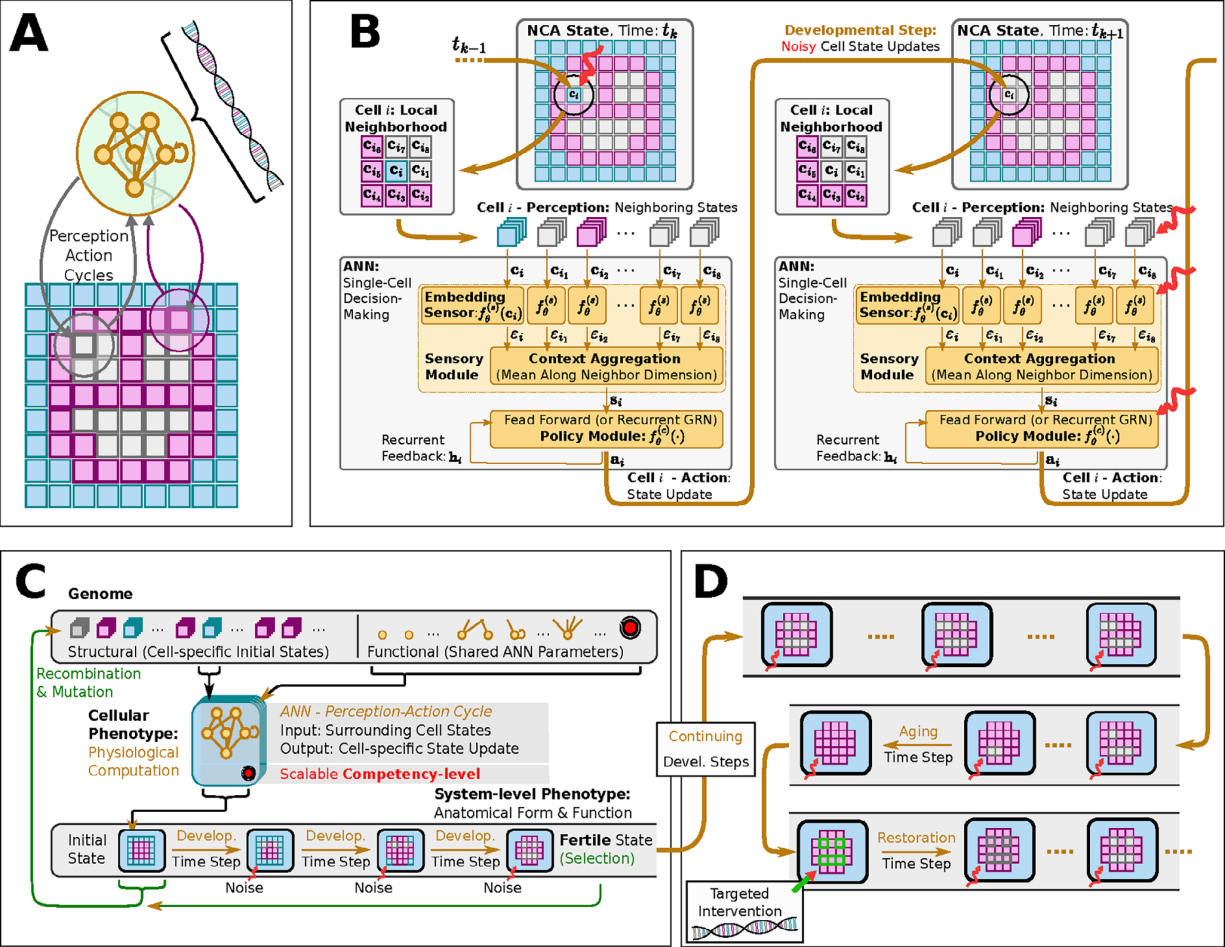
A) Schematic computational model of a biologically inspired multi‐scale competency architecture,^[^ ^23^ ^]^ relying on Neural Cellular Automata^[^ ^44^, ^72^ ^]^ (NCAs) in an evolutionary setting:^[^ ^46^ ^]^ in an evolutionary NCA, a genetic code, i.e., a cells DNA (represented by a string of numerical parameters), is compiled into a uni‐cellular phenotype containing a proto‐cognitive decision‐making center (represented by a tiny recurrent neural network parameterized by the DNA) by which the cellular agent can actively regulate its own numerical cell state based on local measurements of the states of its immediate cellular neighbors on a square grid of cells. In that way, the collective of cells can be trained, or evolved, to perform morphogenesis of a predefined target pattern of expressed cell types (color‐coded in blue, magenta, and white), of, e.g., a smiley‐face pattern (reminiscent of the bioelectric craniofacial prepattern defining the amphibian face.^[^ ^83^ ^]^) B) Detailed information flowchart of uni‐cellular decision‐making of cells on the grid of a NCA, modeling the process of morphogenesis (c.f., (A)): each cell expresses its cell‐type based on local communication protocols with its neighboring cells so the collective of cells self‐assembles a target pattern. This is implemented via subsequent uni‐cellular perception‐action cycles that update each cells respective state on the NCAs grid purely based on local measurements of neighboring cell states. (C) Schematics of the evolutionary process of uni‐cellular competencies that drive the self‐orchestrated morphogenesis of a target pattern (here, of a smiley face). (D) Schematics of long‐term behavior of an NCA evolved in (C) that is not controlled by the evolutionary process. Developmental errors or noise (red wiggly arrows) might lead to a collapse of the target pattern over time, especially long after the selection process occurs, in which genetic material is passed on to the next generation. Following pleiotropic considerations, it was assumed that the long‐term “solutions” of the target morphogenetic state might significantly differ from the target state that the evolutionary process “sees” through selection, simply because different states might be more probable (such as, here, a “red” circle without facial features). Moreover, detecting such deviating pleiotropic defects (missing facial features, in our case) and performing targeted interventions of the most affected cells allow us to “reprogram“ the cellular collective and reset it via targeted interventions ‐ without further optimization or adaptation ‐ so that the tissue auto‐regenerates to the original target pattern.

Recently,^[^ ^46^ ^]^ we quantitatively investigated in silico the influence of varying levels of cellular competency of a multi‐scale competency architecture^[^ ^22^, ^23^ ^]^ on the evolutionary process. We utilized tools from Artificial Life,^[^ ^66^ ^]^ a field of research focused on developing computational and cybernetic models that exhibit life‐like behavior based on ideas derived from biology; one prominent example is Cellular Automata^[^ ^67^, ^68^, ^69^, ^70^ ^]^ (CAs) such as Conway's Game of Life.^[^ ^71^ ^]^ Specifically, we deployed NCAs^[^ ^72^ ^]^ as a model for evolving morphogenesis (see **Figure** **2** A–C): NCAs comprise a regular spatial grid of locally interacting cellular agents which maintain a numerical state vector that is regulated by an internal, cell‐specific Artificial Neural Network (ANN). In that way, the cells of an NCA can be trained to perform a collective self‐orchestrated system‐level task, such as morphogenesis^[^ ^44^, ^45^, ^46^ ^]^ or even collective navigation behavior,^[^ ^56^, ^73^ ^]^ solely via successive perceptions of their local environment, i.e., by reading neighboring cell state information and computing corresponding actions to regulate their own states in accordance with a system‐level agenda.

Thus, NCAs exhibit striking similarities with the genome‐ and collective intelligence‐based multi‐scale competency architectures of biological life :^[^ ^21^, ^23^, ^25^, ^56^, ^74^ ^]^ the instructions for how to grow an organism from the bottom up are encoded in its genome, corresponding to the NCA's (functional) ANN parameters (c.f., Figure 2A). Moreover, through subsequent localized perception‐action cycles of their ANN‐based unicellular agents (c.f., Figure 2B), NCAs model the intercellular communication pathways, intracellular memory, and integrated information processing of the adaptive and flexible internal decision‐making mechanisms of biological cells:^[^ ^75^, ^76^, ^77^, ^78^, ^79^ ^]^ while biological cells regulate their gene expressions via gene regulatory networks, the cells of an NCA regulate their cell types via their internal ANN, and while biological cells utilize bioelectrical and molecular cues for intercellular signaling, cells in an NCA exchange numerical state information in a similar manner. In both cases, such communication pathways are localized between neighboring cells while the collective of cells may still follow a global agenda to self‐orchestrate the process of morphogenesis and thereby give rise to the anatomical form of a system‐level organism.

Building on our previous work,^[^ ^46^ ^]^ we utilize evolutionary algorithms (EAs)^[^ ^80^ ^]^ to optimize the parameters of an NCA so that its cellular agents can reliably self‐assemble a particular spatial target pattern of predefined cell states within a fixed number of developmental steps (c.f., Figure 2C). Inspired by the ability of biological organisms to sustain their physiological integrity for much longer than is necessary for reproduction, we study the long‐term behavior of NCAs by deploying the evolved virtual system for an extended period far beyond the developmental stage at which evolutionary selection occurred. In turn, this may lead to an accumulation of morphological changes that, while not necessarily lethal, can make a multiscale collective increasingly dysfunctional by altering its homeostatic setpoint (c.f., Figure 2D).

Based on such virtual organisms, we here investigate in silico the information‐dynamic implications – using Active Information Storage (AIS),^[^ ^81^ ^]^ Transfer Entropy (TE),^[^ ^82^ ^]^ and Spatial Entropy (SE) – of temporally affected competencies or information loss at the cellular level of a multi‐cellular collective whose parts pursue a system‐level agenda. To systematically interfere with the cellular competencies in our NCA model over time, we deploy four different strategies: We a) increase the misdifferentiation rate of cell‐state expressions; b) reduce the cellular agents' ability to self‐regulate their respective states by corrupting the reliability at which cellular actions are actually considered in the NCA's cell state updates; c) reduce the connectivity between neighboring cells, reminiscent of closing an increasing number of gap junctions (GJs) to neighbors; and d) accumulate genetic damage to the NCA's functional parameters by randomly modifying the corresponding ANN parameters with Gaussian noise successively throughout the lifetime of our virtual organisms.

Thus, we relate degrading competencies and temporal information loss at the cellular level to aging at the system level. Finally, based on the hypothesis of the information‐dynamical implications of aging caused by decaying cellular competencies, we demonstrate – in a minimal in silico model – a reliable method of rejuvenation of facial features, based on targeted interventions at the cellular or organ level.

We suggest that even in the absence of accumulated cellular defects that affect information processing, communication, or genetic material, a biological system that lacks any new goal in the morphospace will begin to degrade anatomically. This is permitted by evolution, as development is typically prioritized over long‐term morphostasis, potentially leaving organisms without a clear anatomical goal after development. Using an NCA‐based in silico model of morphogenesis, we find: 1) Aging arises naturally after development due to the absence of evolved regenerative goals, rather than being caused by specific detrimental properties of developmental programs (e.g., antagonistic pleiotropy or hyperfunction); 2) While cellular misdifferenciation, affected competency and communication pathways, and genetic damage all accelerate aging, they are not its primary causes; 3) Aging correlates with increased active information storage and transfer entropy, while spatial entropy reflects structural loss and accumulated morphological noise; 4) Despite organ loss, corresponding spatial information persists in our cybernetic tissue, indicating that a pattern‐memory of lost structures can be reactivated for organ restoration through targeted regenerative information; 5) Efficient restoration and rejuvenation protocols include regenerative information of differential patterns of affected cells and their neighboring tissue. These findings provide a novel perspective on aging dynamics with significant implications for longevity research and regenerative medicine.

## 2 Experimental Section

### 2.1 Neural Cellular Automaton: A Multi‐Agent Model for Morphogenesis and Aging

CAs were initially introduced by von Neumann to study self‐replicating machines.^[^ ^67^ ^]^ Since then, they had become widely used as simple models for Artificial Life.^[^ ^66^ ^]^ The core concept behind CAs revolved around maintaining a discrete spatial grid of cells, with each individual cell *i* being assigned a binary, numerical, or even vector‐valued state **c**_*i*_(*t*_*k*_) at every time step *t*_*k*_.

The evolution of these cell states over time follows local update rules. Specifically, it was considered that $c_{i}\left(t_{k+1}\right)=u\left(N_{i}\left(t_{k}\right)\right)$, where *u*(·) is a function of the current state of cell *i*, i.e., $c_{i}\left(t_{k}\right)\equiv c_{i_{0}}\left(t_{k}\right)$, and of the states $c_{i_{\nu }}\left(t_{k}\right)$ of its *i*_ν_ = *i*_1_, …, *i*_*N*_ neighboring cells, which were collected in the matrix $N_{i}\left(t_{k}\right)=\left(c_{i_{0}}\left(t_{k}\right),\text{…}\;,c_{i_{N}}\left(t_{k}\right)\right)$.

Despite their typically simple and predefined update rules, CAs often exhibit complex dynamics (c.f., Conway's Game of Life^[^ ^71^ ^]^) and had even been employed for universal computation tasks, as in Wolfram's rule 110.^[^ ^68^, ^69^ ^]^

NCAs^[^ ^72^ ^]^ could be seen as extensions to “traditional” CAs. In this context, the local update rule was replaced with a more flexible ANN, *u*(·) → *f*_θ_(·), where θ denotes the set of trainable parameters of the ANN, *f*_θ_(·) (see, e.g., Appendix A in^[^ ^46^ ^]^). By employing Machine Learning (ML) techniques, NCAs had been utilized to perform tasks such as self‐orchestrated pattern formation^[^ ^44^ ^]^ and the simultaneous co‐evolution of a rigid robot's morphology and controller;^[^ ^56^ ^]^ and they have been proposed as a promising candidate for robust, decentralized controllers of autonomous drug delivery systems.^[^ ^73^ ^]^

An NCA was essentially a grid of interconnected cells, each equipped with an identical ANN that was capable of perceiving the numerical states of its immediate cellular neighbors, $N_{i}\left(t_{k}\right)$, and proposing actions, $a_{i}\left(t_{k}\right)=f_{\theta }\left(N_{i}\left(t_{k}\right)\right)$, to regulate its own cell state, following the equation

(1) $$c_{i}\left(t_{k+1}\right)=c_{i}\left(t_{k}\right)+a_{i}\left(t_{k}\right)+\xi_{c}$$

where possible imperfections in the cell‐specific state updates were also accounted for via a Gaussian noise term of amplitude **ξ**_**c**_.

Thus, the cellular agents of an NCA perceive the numerical states of their immediate respective neighborhoods, $N_{i}\left(t_{k}\right)$, at any given time step, *t*_*k*_, to update their own states (c.f., Figure 2A,B). Importantly, no explicit signature of aging was included in the model, i.e., there was no cell‐internal marker or explicit input to the cellular agents that contains temporal information, other than the self‐regulated cellular states of those agents.

However, being immersed in a dynamical multi‐cellular environment where every part had its own agenda, the cellular agents' state updates could also be utilized for active communicating with their neighbors, following a policy $\pi \left(N_{i}\left(t_{k}\right)\right)\approx f_{\theta }\left(N_{i}\left(t_{k}\right)\right)$. From the perspective of Reinforcement Learning,^[^ ^84^ ^]^ an NCA could thus be considered a trainable multi‐agent system that needs to utilize local communication rules to achieve a target system‐level outcome.

So far, the approach was agnostic to the particular ANN architecture of the update function, *f*_θ_(·). Closely following,^[^ ^46^ ^]^ here, the genuine ANN architecture deployed in our cellular agents was briefly described, which was schematically depicted in Figure 2B: Inspired by,^[^ ^85^ ^]^ the NCA's cells' ANN was partitioned into i) a sensory part, $\varepsilon_{i_{\nu }}=f_{\theta }^{\left(s\right)}\left(c_{i_{\nu }}\left(t_{k}\right)\right)\in R^{s}$, preprocessing each neighborhood cell state separately into a respective sensor embedding, which were collected in the matrix $E\left(N_{i}\left(t_{k}\right)\right)=\left(\varepsilon_{i_{0}}\left(t_{k}\right),\text{…}\;,\varepsilon_{i_{N}}\left(t_{k}\right)\right)$. These sensor embeddings are then ii) averaged across all neighbor embeddings into a single context vector $s_{i}\left(t_{k}\right)=\frac{1}{N+1}\sum_{\nu =0}^{N}\varepsilon_{i_{\nu }}\left(t_{k}\right)\in R^{s}$. A subsequent iii) controller ANN, $f_{\theta }^{\left(c\right)}\left(\cdot \right)$, potentially with recurrent feedback connections, eventually outputs the cell's action, $a_{i}\left(t_{k}\right)=f_{\theta }\left(N_{i}\left(t_{k}\right)\right)=f_{\theta }^{\left(c\right)}\left(s_{i}\left(t_{k}\right)\right)$ based on the cell‐specific context vector **s**_*i*_(*t*_*k*_). Notably, for the controller module, so‐called RGRN architectures were utilized^[^ ^46^ ^]^ that were inspired by a combination of *Recurrent* ANNs (RNNs)^[^ ^86^ ^]^ and *Gene Regulatory Networks* ^[^ ^87^ ^]^ (see Appendix A in^[^ ^46^ ^]^ for details).

Analogous to,^[^ ^46^ ^]^ morphogenesis was modeled by employing NCAs on a 2D *N*_*x*_ × *N*_*y*_ square grid with the objective that all cells of the grid assume a cell‐specific predefined target cell type, ${\hat{g}}_{i}$, after a fixed number of *t*_D_ developmental time steps, starting from an initial cell state configuration **c**_*i*_(0), The first *N*_G_ elements, $g_{i}\left(t_{k}\right)=\left(c_{i1}\left(t_{k}\right),\text{…}\;,c_{iN_{G}}\left(t_{k}\right)\right)$, of an NCA's $N_{C}$‐dimensional cell state $c_{i}\left(t_{k}\right)=\left(c_{i1}\left(t_{k}\right),\text{…}\;,c_{iN_{C}}\left(t_{k}\right)\right)$ were assigned as indicators for expressing one of $1,\text{…}\;,N_{G}$ discrete cell types, while the remaining $N_{H}=\left(N_{C}-N_{G}\right)$ elements of the cell state represent its hidden states, $h_{i}\left(t_{k}\right)=\left(c_{i(N_{C}-N_{H})}\left(t_{k}\right),\text{…}\;,c_{iN_{C}}\left(t_{k}\right)\right)$. While the full $N_{C}$‐dimensional cell state vectors **c**_*i*_(*t*_*k*_) could be utilized for intercellular communication by the NCA, a cell's “type”, *g*_*i*_(*t*_*k*_), is now defined as the index, $1,\text{…}\;,N_{G}$, of the maximum element of its indicator vector **g**_*i*_(*t*_*k*_):

(2) $$g_{i}\left(t_{k}\right)=\underset{g\in R^{N_{G}}}{\arg\max}\left(g_{i}\left(t_{k}\right)\right)$$

For an NCA to assemble a predefined target pattern of *N*_j_ = *N*_*x*_ × *N*_*y*_ target cell types $\hat{g}=\left\{{\hat{g}}_{1},\text{…}\;,{\hat{g}}_{N_{j}}\right\}$, a suitable set of NCA parameters must thus be found that minimizes the deviation of all actual cell types $g_{i}\left(t_{D}\right)$ and the desired ones ${\hat{g}}_{i}$ after $t_{D}$ developmental time steps. Below, an evolutionary algorithm was introduced to evolve suitable sets of NCA parameters that maximize a fitness score based on comparing the “final” cell types of the NCA, $g_{i}\left(t_{D}\right)$, after the developmental stage to the predefined target cell types ${\hat{g}}_{i}$. In this context, the NCA parameters thus correspond to the virtual organism's genotype, while the grid of final cell types represent the system‐level phenotype that was seen by the selection mechanism of the evolutionary process (c.f., Figure 2C).

### 2.2 Neuroevolution of NCAs: An Evolutionary Algorithm Approach to Morphogenesis

Evolutionary Algorithms (EAs) were heuristic optimization techniques designed to maintain and optimize a set, or population, $X=\{x_{1},\text{…}\;,x_{N_{P}}\}$, comprising parameters or individuals, $x_{j}\in R^{X}$, over successive generations in order to maximize an objective function or fitness score, $r\left(x_{j}\right):R^{X}\to R$. Drawing inspiration from the principles of natural selection and biological life's DNA‐based reproduction mechanisms, EAs predominantly select high‐fitness individuals for reproduction, and utilize crossover and mutation operations to generate new offspring by merging the genomic material of two high‐quality individuals from the current population, $x_{o}=x_{j}⨁x_{k}$, and occasionally mutating offspring genomes, **x**_*o*_ → **x**_*o*_ + ξ_**x**_, by adding typically Gaussian noise to the parameters; the ⨁ symbol signifies a genuine merging operation of two genomes, which may vary according to the specific EA implementation. Thus, populations of individuals were directed toward high‐fitness regions within the parameter space $R^{X}$, typically across numerous generations of successive selection and reproduction cycles.^[^ ^46^ ^]^

Closely following the previous work^[^ ^46^ ^]^ and in contrast to many “traditional” EA use‐cases, a distinguish was explicitly made between the genotypic parameters, **x**_*j*_, and the corresponding phenotypic realizations, **p**_*j*_. More specifically, we utilize NCAs (c.f., Section 2.1) to model the biologically inspired developmental layer^[^ ^23^ ^]^ in between genotypes and phenotypes, **x**_*j*_ → **p**_*j*_, akin to the process of morphogenesis: Here, a genotype, **x**_*j*_, corresponds to a particular realization *j* of an NCA's parameters, comprising the set of initial cell states $x_{j}^{\left(S\right)}={\left\{c_{i=(1,\text{…}\;,N_{j})}\left(0\right)\right\}}_{j}$ and the corresponding ANN parameters $x_{j}^{\left(F\right)}=\theta_{j}$, such that

(3) $$x_{j}=x_{j}^{\left(S\right)}\cup x_{j}^{\left(F\right)}=\left({\left\{c_{i}\left(0\right)\right\}}_{j},\theta_{j}\right),$$

where a distinguish was explicitly made between structural (S) and functional (F) genes. Equation (1) was then employed for *t*_D_ developmental steps to successively transform the NCA's cell states from an initial state into a “final” set of cell types {*g*_*i*_(*t*_D_)}_*j*_ on the NCA's grid. This mature phenotype,

(4) $$p_{j}={\left\{g_{i}\left(t_{D}\right)\right\}}_{j}$$

represents a 2D tissue of cells, and is the input to the fitness score based on which the EA selects: As illustrated in Figure 2C, optimization was performed for a fitness score in the phenotype space rather than the genotype space, *r*(**x**_*j*_) → *r*(**p**_*j*_), while still performing genetic recombination and mutation in the genotype space.

Let's recall: the goal was to achieve morphogenesis of a 2D spatial tissue of cell types, **p**_*j*_, that optimally resembles a predefined target pattern, $\left\{{\hat{g}}_{1},\text{…}\;,{\hat{g}}_{N_{j}}\right\}$, of a total of *N*_*j*_ cells on an *N*_*x*_ × *N*_*y*_ square grid of an NCA. Moreover, the aim was to derive a virtual organism with this capability by modeling a biologically inspired evolutionary process with EAs to evolve a suitable set of NCA parameters (c.f., Figure 2 (C)). Thus, the phenotype‐based fitness score was introduced, *r*(**p**_*j*_), as^[^ ^46^ ^]^

(5) $$r\left(p_{j}\right)=\left(2n_{j}^{\left(G\right)}-N_{j}\right)+r_{T}\;n_{j}^{\left(T\right)}-r_{S}\;n_{j}^{\left(S\right)}$$

where $n_{j}^{\left(G\right)}$ counts the number of correctly assumed cell types after *t*_D_ developmental steps (where $g_{i}\left(t_{D}\right)={\hat{g}}_{i}$), $n_{j}^{\left(T\right)}$ counts the number of developmental steps at which the target pattern is realized entirely (whenever $g_{i}\left(t_{k}\leq t_{D}\right)={\hat{g}}_{i}$ for all *i*), and $n_{j}^{\left(S\right)}$ counts pairs of successive time steps, (*t*_*s*_, *t*_*s* + 1_) ⩽ *t*_D_, without cell type updates in the entire tissue (where *g*_*i*_(*t*_*s* + 1_) = *g*_*i*_(*t*_*s*_) for all *i*).

Thus, an evolutionary process was designed that primarily selects for NCAs that assume the correct cell type pattern after (precisely) *t*_*D*_ developmental steps and reinforces NCAs (by a factor of *r*_*T*_ in the fitness score) that prematurely achieve and maintain the target pattern. To avoid developmental stagnation and increase the EA's performance, the fitness score of NCAs was specifically discounted with static behavior by *r*_*S*_. A fitness score of *N*_*j*_ = *N*_*x*_ × *N*_*y*_ indicated that the problem is solved.

Although the above framework^[^ ^46^ ^]^ could be utilized in conjunction with any black‐box evolutionary or genetic algorithm, the well‐established Covariance Matrix Adaptation Evolutionary Strategy (CMA‐ES)^[^ ^80^ ^]^ to be a viable choice for our purposes.

### 2.3 Information‐Theoretic Analysis: Active Information Storage, Transfer Entropy, and Spatial Entropy on the NCA

Information theory^[^ ^88^ ^]^ offers valuable insights into the dynamics of complex systems. In order to understand the NCA's information dynamics during aging, three key information‐theoretic metrics were used to examine the information dynamics: active information storage,^[^ ^81^ ^]^ transfer entropy,^[^ ^82^ ^]^ and the spatial entropy.

Active Information Storage (AIS) quantified the amount of information from an agent's past that was pertinent to predicting its future state. Specifically, AIS refers to the portion of stored information currently utilized for determining the agent's next state.^[^ ^81^ ^]^ Mathematically, the AIS of an agent *Q* was expressed as the local mutual information between its semi‐infinite past $q_{n}^{\left(k\right)}$ as *k* → ∞ and its next state *q*_*n* + 1_ at time step *n* + 1:

(6) $$a_{Q}\left(n+1\right)=\lim_{k\to \infty }\log_{2}\frac{p(q_{n}^{\left(k\right)},q_{n+1})}{p\left(q_{n}^{\left(k\right)}\right)p\left(q_{n+1}\right)}$$

Here, *a*_*Q*_(*n*, *k*) is an approximation with history length *k*. The time‐averaged value, weighted by the distribution of $\left(q_{n}^{\left(k\right)},q_{n+1}\right)$, is denoted as *A*_*Q*_(*k*) = 〈*a*_*Q*_(*n*, *k*)〉. In this study, the local was calculated AIS across the states of the cells.

Transfer Entropy (TE) measures the information transferred from a source agent to a destination agent that was not contained in the past of the destination agent. The local TE concept introduced by Lizier was employed.^[^ ^81^ ^]^ The local TE from a source agent *Z* to a destination agent *Q* was defined as the local mutual information between the previous state of the source *z*_*n*_ and the next state of the destination agent *q*_*n* + 1_, conditioned on the semi‐infinite past of the destination $q_{n}^{\left(k\right)}$ (as *k* → ∞):

(7) $$t_{Z\to Q}\left(n+1\right)=\lim_{k\to \infty }\log_{2}\frac{p(q_{n+1}|q_{n}^{\left(k\right)},z_{n})}{p(q_{n+1}|q_{n}^{\left(k\right)})}.$$

The transfer entropy *T*_*Q*_(*n*, *k*) was the local TE averaged over time, denoted as *T*_*Q*_(*k*) = 〈*t*_*Q*_(*n*, *k*)〉, where *t*_*Q*_(*n*, *k*) was an approximation with history length *k*. Unlike mutual information, which measures static correlation, transfer entropy captures the dynamic, directional flow of information within the agent network.

Spatial Entropy (SE) measured the randomness or disorder within a spatial distribution of states. It provided insight into the complexity of spatial patterns in a system, such as an NCA with multiple states. In this study, SE was computed by evaluating the entropy of the distribution of cell states across the grid at each time step. Formally, the spatial entropy *H* at time step *n* is defined as:

(8) $$H\left(n\right)=-\sum_{s\in S}p\left(s\right)\log_{2}p\left(s\right)$$

where *S* is the set of possible states, and *p*(*s*) is the probability of state *s* occurring in the grid at time step *n*. This measure provided an indication of the level of unpredictability or complexity in the spatial arrangement of cell states. In this article, the spatial entropy for the states of the NCA was calculated across different time steps.

## 3 System

Following our previous study,^[^ ^46^ ^]^ we utilize EAs to evolve NCAs to perform morphogenesis tasks. Each cell in our NCA implementation is equipped with a gene regulatory network‐inspired recurrent ANN, conceptually substituting the intricate internal decision‐making competencies of biological cells (see Figure 2B for a visualization and Appendix A in^[^ ^46^ ^]^ for numerical details). In other words, each cell in the NCA is equipped with an information processing pipeline that transforms sensory input of its local environment in the NCA's grid into control instructions to regularize its own cell type. If done in a coordinated way, these local individual cell‐state updates guide the formation of a mature system‐level phenotype ‐ in our case of a target tissue ‐ over successive developmental steps.

More specifically, we utilize *N*_*x*_ × *N*_*y*_ = 16 × 16 cells on an NCA with fixed boundary conditions (see Section 2.1 and Appendix C in^[^ ^46^ ^]^) and train it analogously to^[^ ^46^ ^]^ via evolutionary algorithms (see Section 2.2) to perform morphogenesis ‐ i.e., self‐orchestrated tissue growth ‐ of a 16 × 16 smiley‐face pattern in a predefined number of *t*_D_ = 35 developmental steps (see Figure 2C for an illustration of the evolutionary process); the smiley‐face‐pattern is reminiscent of the bioelectric craniofacial prepattern that establishes the rough outlines of the amphibian face during early development.^[^ ^89^ ^]^

The target tissue consists of *N*_G_ = 3 distinct cell types: one cell type for the background cells, one for the face cells, and a single cell type for the facial organs, i.e., for the eyes and the mouth (c.f., blue‐, magenta‐, and white‐colored cells in Figure 2A). Moreover, we allow the NCA to utilize *N*_H_ = 1 hidden channel of its *N*_C_ = 4 cell state channels for unconstrained intercellular communication. In cell‐state updates, given by Equation (1), we utilize a noise level of ξ_**c**_ = 0.125, limit the numerical value of the proposed action **a**_*i*_(*t*_*k*_) to *l*_**a**_ = [− 1, 1], and clip the cell‐states post‐update **c**_*i*_(*t*_*k* + 1_) to *l*_c_ = [− 3, 3] (see Appendix C and^[^ ^46^ ^]^ for more details on our NCA implementation).

**Figure 3:**
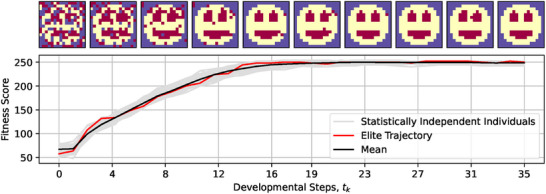
Typical fitness score evaluations during *t*_D_ = 35 developmental steps of morphogenesis of 50 statistically independent runs of an NCA that has been evolved to self‐assemble a 16 × 16 smiley‐face pattern. Example NCA states from the individual with the highest developmental fitness score (c.f., red “elite trajectory” in bottom panel) are presented throughout the developmental phase as temporal snapshots corresponding to the ticks of the horizontal axis; background‐, facial‐, and internal organ cells (of eyes and mouth) are colored purple, yellow, and red, respectively.

Our NCAs transform an embryonic genotype, **x**_*j*_, into a mature phenotype, **p**_*j*_, and we here quantify an NCA's performance by evaluating the corresponding fitness score, *r*(**p**_*j*_), defined by Equation (5) with respect to the 16 × 16 smiley‐face pattern over the NCA's entire life‐span of *t*_D_ developmental steps. As illustrated in **Figure** **3**, a successfully evolved NCA maximizes the fitness score to *r*_max_ = 256, exhibiting 16 × 16 correctly expressed cell types after *t*_*k*_⪆20 time steps, and then maintaining the target pattern until *t*_*k*_ = *t*_D_ (see Section 2.2 for details on the evolutionary process).

Notably, the cellular agents do not perceive any information about the current nor the final fitness score of their corresponding NCA. The fitness score is only utilized by the EA to optimize the NCA's parameters over evolutionary time‐scales. Moreover, there is no contribution either to the fitness score or to the NCA's ANN sensory inputs that would provide the system with explicit temporal information indicating the “age” of any part of the virtual organism, or even a notion of “time” in general. Thus, the self‐orchestrated pattern formation of the here ‐ and previously^[^ ^46^ ^]^ ‐ investigated NCAs is fully driven via emergent (i.e., evolved) intercellular communication rules and intracellular decision‐making.

## 4 Computational Results

### 4.1 Aging as a Loss of Goal‐Directedness: Organism Learned Development During Evolution, not to Maintain Anatomical Homeostasis After Development

We aimed to assess whether the self‐regulatory behavior of biological systems that drive morphogenesis induces aging‐like phenomena at long time‐frames. To this end, we use NCAs evolved in silico to model the process of morphogenesis and then deploy their self‐regulatory dynamics for much longer times compared to the reproduction stage during evolution (i.e., when selected for reproduction). We monitor and analyze the NCAs morphological integrity throughout their lifetimes and compare such long‐term morphological trajectories with biological aging. The evolutionary process described in Sections 2.2, 3 explicitly selects for optimal fitness scores after *t*_D_ developmental steps, which corresponds to the fertility age of real‐world organisms. However, this implies that the behavior of an NCA with a potentially much longer lifetime of *t*_A_ > *t*_D_, and particularly *t*_A_ ≫ *t*_D_, is not subject to selection and may fundamentally be considered ill‐defined or ambiguous.

**Figure 4:**
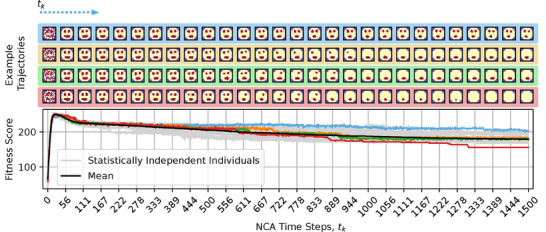
Same as Figure 3, but evaluated for *t*_A_ = 1500 developmental (or NCA‐) time steps, furthermore displaying multiple example trajectories of NCA‐states (color‐coded): The individual emphasized in blue maintains an approximate smiley‐face pattern for exceptionally long times, and thus displays the highest long‐term fitness score. The orange‐ and green‐emphasized individuals lose, respectively, either a left or right eye at *t*_*k*_ ≈ 600 − 700 steps, and both eyes at *t*_*k*_ ≈ 900 steps, but maintain their mouth for long times; these individuals display close‐to‐average long‐term fitness scores. The red‐emphasized individual eventually loses the mouth, thus displaying the lowest long‐term fitness score among the presented trajectories. The spherical face structure seems incredibly robust.

To demonstrate this, we deploy the NCA solution that has been evolved to self‐assemble a 16 × 16 smiley‐face pattern during a morphogenesis phase of *t*_D_ = 35 developmental steps (c.f., Figure 3) for a much longer lifetime of up to *t*_A_ = 1500 time steps, notably without any further optimization considering the extended lifetime. From the results presented in **Figure** **4**, we learn that after an initial rapid rise in the fitness score during the developmental phase, the NCA's long‐term fitness score gradually declines, primarily due to the loss of different internal organs such as one or both eyes and, occasionally, the mouth. Moreover, this is not only a feature of the particular NCA demonstrated here, but is a systematic phenomenon of the presented framework.

Thus, we argue that the biologically‐ubiquitous self‐regulatory behavior of a substrates agential parts may induce system‐level dynamics that resemble the process of biological aging if applied on timescales that exceed development. The organisms learned during evolution with development as the primary goal; once they reached that goal, we observed that they don't regenerate by themselves and slowly show signs of morphological deterioration, e.g., aging. In this sense, aging can be seen primarily as a loss of goal‐directedness, the goal of development being different from the goal of maintenance of the anatomy over timescales exceeding development. This view on aging is related to programmatic theories of aging like antagonistic pleiotropy or hyperfunction theory^[^ ^11^, ^16^, ^17^ ^]^ that state that what is good for development could ultimately cause aging. In our framework, the lack of goal‐directedness after development may give rise to informational antagonistic pleiotropy (or hyperfunction) as cells may lose some developmental policies after reaching the target morphology. However, our framework goes beyond antagonistic pleiotropy by enabling the deliberate re‐targeting of the morphogenetic setpoint in an aging tissue toward new anatomical goals. In turn, a continually regenerative setpoint – e.g., by reactivating developmental pathways – may reverse aging, as we demonstrate in silico in Section 4.4.

### 4.2 Impact of Defects of Cellular Information Processing at Different Levels on the Rate of Aging in a Multi‐Scale Competency Architecture

To determine how the perception‐action cycles of the unicellular agents of a collective multi‐cellular organism influence, at least in part, the rate of morphological decline, we here investigate the effects of an NCA's system parameters on its long‐term morphological integrity. Based on the NCA system defined in Section 3, we identify four key mechanisms involved in the corresponding uni‐cellular perception‐action cycles affecting morphological integrity: a) cell‐specific differentiation reliablility, b) reliable state updates, c) reliable communication between neighbors, and d) persistent genetic encoding. Our framework allows us to selectively control these corresponding processes (a–d) and explicitly corrupt them independently over the lifetime of the virtual organism. Thereby we demonstrate the severe effects of these processes on the rate of morphological aging.

**Figure 5:**
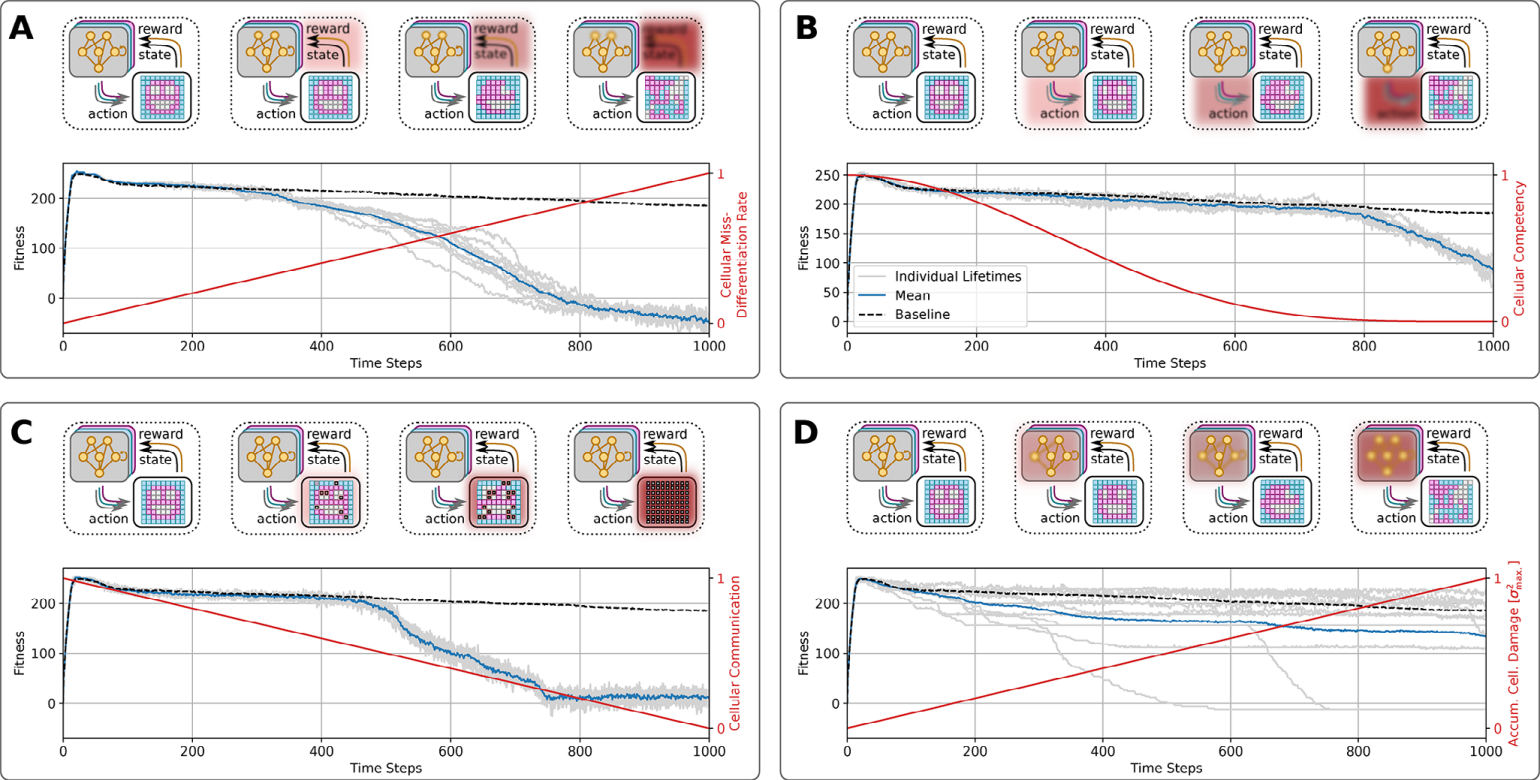
Panels (A–D): Schematic visualization of the amplified damaging processes to cellular competencies corresponding to Sections 4.2.1, 4.2.2, 4.2.3, 4.2.4, respectively. A) The fitness over the lifetime of the 16 × 16 smiley‐face NCA discussed in Section 3 where the noise level for cell‐state updates is linearly increased from ξ_*c*_(*t*_*k*_ = 0) = 0 to ξ_*c*_(*t*_A_) = 1 (emphasized by red line), for *t*_A_ = 1000 time steps *t*_*k*_; gray lines show statistically independent trajectories of individual lifetimes of the NCA, the blue line represents the corresponding ensemble mean, and the black‐dashed line is the baseline mean fitness without corrupted competencies (c.f., Figure 4). (B): similar to (A) but corrupting cellular competencies via a successively decreasing decision‐making probability *P*_D_(*t*_*k*_ = 0) = 1 to *P*_D_(*t*_A_) = 0 following the functional form $P_{D}\left(t_{k}\right)=\sin{\left[\frac{\pi }{2}\left(1-\frac{t_{k}}{t_{A}}\right)\right]}^{4}$ (see red line). (C): similar to (A, B) but permanently corrupting the intercellular communication pathways by successively and randomly disabling selected communication channels (reminiscent of gap‐junctions^[^ ^22^ ^]^) between neighboring cells by linearly decreasing the gap‐junction prohibiting probability from *P*_GJ_(*t*_*k*_ = 0) = 1 to *P*_GJ_(*t*_A_) = 0 (see text and red line). (D): similar to (A–C) but accumulating genetic damage in the unicellular decision‐making machinery, i.e., corrupting intracellular information processing and decision‐making by adding zero‐centered Gaussian noise of standard deviation σ_**x**_ = 10^−2^ at every time step to the cells' ANN parameters (which is equivalent to ANN parameters corrupted by $\sigma_{x}^{2}\left(t_{k}\right)=t_{k}\sigma_{x}^{2}$ at time‐step *t*_*k*_ with $\sigma_{\max}^{2}=\sigma_{x}^{2}\left(t_{A}\right)$). In (A–D), the top panels schematically illustrate the perception‐action cycles of every cell over the lifespan of the NCA; the particular processes affecting the cellular competencies, thus enhancing the rate of degradation of the target pattern in each panel (A–D), are emphasized from left to right by red blurry blocks: this involves (A) increasing misdifferentiation and misperception of cell states by neighbours, (B) increasing unreliable cell decisions directly affecting the NCA's action output, (C) successively corrupted intercellular communication channels directly affecting the exchange of information between neighbors, and (D) accumulation of genetic damage by successively diffusing the cells' ANN parameters directly affecting the unicellular decision‐making machinery.

In the following sections, we therefore study the factors accelerating aging, not the cause of aging per se that we see primarily as a loss of goal‐directedness. We introduce these known aging mechanisms (a–d), respectively, based on and deployed to the biologically motivated unicellular competencies in our NCA system. We then demonstrate and discuss the corresponding implications on enhanced rates of morphological aging in long‐term simulations of such virtual organisms. We illustrate the respective results in a compact form in **Figure** **5**A–D, and continue below with an information‐theoretic analysis of these processes.

#### 4.2.1 Cellular Differentiation

One critical parameter in our system is the noise level ξ_**c**_ utilized to corrupt the NCA's update step given by Equation (1). While a moderate noise level can help an evolutionary process to identify robust NCA parameters that solve a specific morphogenesis task; but if the selective noise level is too low, the evolutionary process will in our case most likely identify a direct encoding of the target pattern, largely neglecting cellular competencies in the pattern formation task.^[^ ^46^ ^]^ In turn, a too‐large noise level will not only prevent the evolutionary process from finding a good solution to the morphogenesis task but, in general, will drastically limit the cell specification capacities of the NCAs cell states. Therefore, noisy cell state updates in our system could be seen as the accumulation of misdifferentiated cells, ultimately corrupting the state perceptions of neighboring cells on the NCA and thereby creating a default of cellular communication (a hallmark of aging) similarly to.^[^ ^6^, ^90^ ^]^ This mechanism accelerates aging but is not a cause per se.

We argue that an increasing noise level ξ_**c**_ → ξ_**c**_(*t*_*k*_) over the lifetime of such an NCA‐akin organism successively affects its morphological integrity. To demonstrate this, we thus utilize the pre‐evolved NCA from above (c.f., Figures 3, 4) and deploy it for an extended lifetime of *t*_A_ = 1000 timesteps while successively increasing the noise level linearly from ξ_**c**_(*t*_*k*_ = 0) to ξ_**c**_(*t*_*A*_) = 1. The results depicted in Figure 5A show that while the correspondingly affected virtual organisms initially follow their unaffected cousins (c.f., Figures 3, 4 and black‐dashed line in Figure 5A), after a particular duration (specific to the particular NCA solution and noise schedule ξ_**c**_(*t*_*k*_)) the fitness score rapidly drops at *t*_*k*_ ≈ 380 − 400, emphasizing an enhanced rate of degradation of the target tissue compared to the baseline with constant noise ξ_**c**_ = 0.125.

Noise influences the dynamical cell states in our NCA experiments. These states are the inputs and outputs of the virtual tissues' ANN augmented cells, and represent dynamic variables of the self‐regulatory NCA dynamics, with attractor states or instabilities. We thus interpret the effect discussed here as cellular misdifferentation‐enhanced morphological aging. Understanding such “run‐away” arguments in the “biological software” of cell state expressions, or instabilities in the context of competent tissues, seems central to understanding aging.

#### 4.2.2 Cellular Competency

Next, we directly manipulate the cellular decision‐making competency ‐ the reliability with which uni‐cellular agents can regulate their own cell states within a tissue (e.g., regulative development and regenerative tissue allostasis). Here, we investigate whether degrading unicellular competencies throughout the lifetime of an organism enhances morphological aging: while young, healthy cells are highly reliable in their (re)actions to external perturbations, especially during development, the reliability of such cell decisions might be strongly affected over the lifetime of an organism, inducing morphological decline. To this end, and following,^[^ ^46^ ^]^ we define the decision‐making as a probability *P*_D_ at which proposed actions of unicellular agents are actually forwarded to the environment at a given time step. More specifically, at every instance in time *t*_*k*_ we draw a uniform random number *r*_*i*_ ≈ rand ∈ [0, 1] for every cell *i* = 1, … , *N* in the tissue. Only if *r* ⩽ *P*_D_ do we consider the cell's action *a*_*i*_(*t*_*k*_) in the NCA's update step, described by Equation (1); otherwise we omit it by setting *a*_*i*_(*t*_*k*_) = 0. In that way, we can scale the average reliability of decision‐making of all cells in the tissue, i.e., while *P*_D_ = 1 corresponds to fully reliable decision‐making of every cell at every time step, *P*_D_ = 0 parameterizes fully passive cells which can't make decisions at all.

Here, we utilize *P*_D_ → *P*_D_(*t*_*k*_) as decreasing cellular competency by transforming *P*_D_(*t*_*k*_ = 0) = 1 to *P*_D_(*t*_A_) = 0 in *t*_A_ = 1000 time steps following the functional form $P_{D}\left(t_{k}\right)=\sin^{4}\left(\frac{\pi }{2}\left(1-\frac{t_{k}}{t_{A}}\right)\right)$ (see Figure 5 B). This increasingly corrupts cellular competencies by successively decreasing the reliability at which every unicellular agent in the grid can regularize, and thus “correct” their states against noise or faulty cell state configurations.

The results of Figure 5B reveal that the fitness score of NCAs whose cellular competencies are gradually affected remains similar to the baseline fitness scores of the unperturbed case (c.f., Figures 3, 4). But eventually, at *t*_*k*_ ≈ 800, the small but finite noise of ξ_**c**_ = 0.125 used in our simulations leads to accumulated damage in the NCA's states that is catastrophic for its morphological integrity, thus destroying the target morphology via diffusive processes.

Thus, decreasing cellular decision‐making competencies in a noisy collective organism necessarily lead to morphological aging effects, caused by unreliability in the “biological cell‐state reconfiguration software”. However, the rate at which these decreasing cellular competencies lead to significant aging effects is the result of an intricate interplay between the actual noise in the system and the robustness of the unicellular agents to persist against accumulating noise in their respective state configurations.

#### 4.2.3 Cell–Cell Communication

Another necessary feature of systems with decentralized collective decision‐making is reliable communication between agents. Similar to biological tissue, the cells of an NCA are integrated into a lattice of functionally homogeneous cells that only diverge in their respective cell state history. However, their neighbor coordination typically remains fixed.^[^ ^44^, ^46^, ^72^ ^]^ Here, we specifically manipulate the connectivity between the unicellular agents in the NCA's grid by blocking an increasing number of communication channels between neighboring cells as time proceeds. This approach is biologically inspired by cells closing their gap junctions (GJs) to their neighbors, which is known in vivo to shift cells from cooperatively working on large morphogenetic setpoints (organ maintenance) toward individual goals appropriate to unicellular organisms such as proliferation and migration.^[^ ^22^, ^91^, ^92^, ^93^ ^]^ It is also related to altered cell–cell communication found during aging.^[^ ^6^ ^]^ Indeed, aging involves deficiencies in neural, neuroendocrine, and hormonal signaling pathways^[^ ^94^ ^]^ as well as alterations in the bidirectional communication between human genome and microbiome. Chronic inflammation is also part of this altered cellular communication.^[^ ^6^ ^]^

We achieve this technically by omitting the corresponding input state **c**_*ij*_ = **0** of a blocked connection, or GJ from cell *j* to cell *i* (see Figure 2B). More specifically, we utilize a probability *P*_GJ_(*t*_*k*_) which defines the fraction of GJs that should remain open for each cell in the tissue at a given time *t*_*k*_. In turn, if for a particular cell *i*, the fraction of connections to its neighbors exceeds *P*_GJ_(*t*_*k*_) at time *t*_*k*_, we henceforth randomly block another one of its *i*_ν_ = *i*_1_, … , *i*_*N*_ still‐connected GJs (once closed, GJs remain closed in our case); see Section 2.1 and^[^ ^46^ ^]^ for more details on our definition of cellular neighborhoods.

By linearly transforming *P*_GJ_(*t*_*k*_ = 0) = 1 to *P*_GJ_(*t*_A_) = 0, we successively isolate the unicellular agents from their host tissue by blocking an increasing number of their GJs until, eventually at time *t*_A_ = 1000, all cells are effectively isolated from their surroundings. In Figure 5C, we demonstrate the effects of this approach: Again, the self‐orchestrated morphogenetic program can withstand the effects of increasingly corrupted competencies for a surprisingly long time. However, at *t*_*k*_ ≈ 500, corresponding to *P*_GJ_ = 0.5 (half the GJs of every cell being closed), the effects of the removed communication channels can be observed as a resulting rapid drop in the phenotypical fitness scores caused by a rapid degradation of the target pattern.

Thus, decreasing the ability of cells to communicate with their neighbors leads to enhanced effects of morphological aging if a threshold of GJs is closed.

#### 4.2.4 Accumulation of Genetic Damage

Another potential source for self‐induced aging is accumulating damage to the cells' genetic material via successive mutations of the functional genome **x** ^(F)^ → **x** ^(F)^(*t*_*k*_) over the lifetime of the virtual organism: To this end, we utilize the pre‐evolved NCA solution discussed above (c.f., Figures 3, 4), but at every time step *t*_*k*_ > 0, we successively manipulate the cells' ANN parameters throughout their lifetime. This directly introduces accumulating genetic mutations to the self‐regulatory mechanisms of the NCA, causing potential dysfunctional decision‐making behavior at the uni‐cellular level, and correspondingly leading to enhanced morphological decline. More specifically, we add zero‐centered normal distributed noise $N(0,\sigma_{x})$ of standard deviation σ_*x*_ = 0.01 to the functional genes of the NCA, **x** ^(F)^(*t*_*k* + 1_) = **x** ^(F)^(*t*_*k*_) + δ_**x**_, where $\delta_{x}\approx N\left(0,\sigma_{x}\right)$. The mutated functional genes **x** ^(F)^(*t*_*k*_) are then mapped to the ANN parameters θ → θ(*t*_*k*_) of the unicellular agents' controllers at every time step *t*_*k*_, thus directly affecting the intracellular decision‐making machinery of the NCA.

From the results depicted in Figure 5D we can learn several things: First, the evolved NCA solutions seem incredibly resistant to accumulating genetic damage in the ANN parameters, which is reflected by the fact that most of the deployed simulations (with statistically independent mutations) retain high‐fitness target patterns for the entire simulated lifetime of the NCA of *t*_A_ = 1000 timesteps. Second, and in contrast to Sections 4.2.1, 4.2.2, 4.2.3, this leads to large qualitative deviations in individual behavior of an ensemble of statistical independently mutated NCA solutions: some solutions die off early and obtain vanishing fitness scores, others degrade rather continuously with a spread‐out fitness score of ≈100, and many solutions still maintain their integrity although accumulating significant genetic damage of $\sigma_{\max}^{2}=t_{A}\sigma_{x}^{2}=0.1$ in total.

Thus third, while many mutations appear neutral in our framework, critical mutations to specific genes might induce detrimental modifications to the behavior of the unicellular agents (if they occur at the “correct” moment), typically causing a rapid degradation of the target pattern of the affected NCA as reflected by significant drops in the fitness scores of the corresponding virtual tissue. Notably, due to technical reasons, we mutate the functional genome of every agent homogeneously, thus retaining a functionally homogeneous distributed decision‐making in the NCA, which nevertheless deviates functionally from the baseline at *t*_*k*_ = 0. Potentially intriguing effects based on heterogeneous mutations (and thus heterogeneous agents in the NCA) are out of the scope of this work, and we leave a more thorough investigation to a future contribution.

### 4.3 The Acceleration of Aging is Linked to Increase in AIS and TE, While Spatial Entropy Revealed Two Different Kind of Aging: Loss of Structure and Proliferation, and Accumulation of Morphological Noise

**Figure 6:**
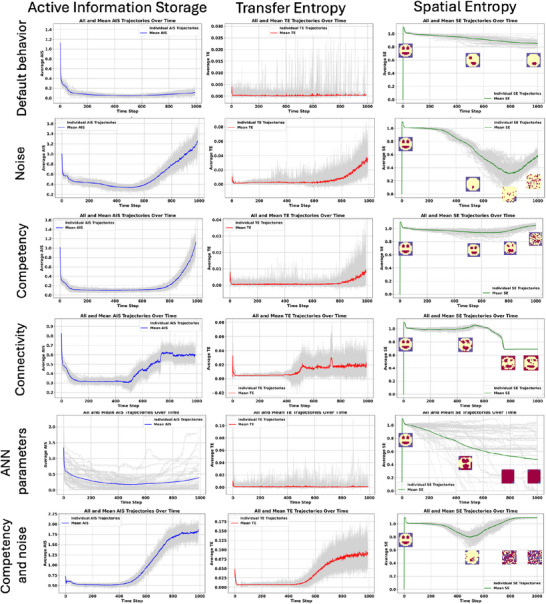
Active information storage (AIS), transfer entropy (TE) and spatial entropy (SE) during development and aging for six conditions: default behavior, noise, competency, connectivity, the alterations of ANN parameters, and noise and competency. Left: blue is the mean of the AIS for the 50 organisms, light grey is the individual AIS mean per organism. Middle: red is the mean of the TE for the 50 organisms, light grey is the individual TE mean per organism. Right: green is the mean of the SE for the 50 organisms, light grey is the individual SE mean per organism. At different stages, we show the states of the organism.

In order to understand the information dynamics of aging and the cellular information processing in a competent tissue, we applied information‐theoretic measures such as Active Information Storage (AIS), Transfer Entropy (TE), and Spatial Entropy (SE), to the states of the organism (see **Figure** **6**).

In the default behavior case, without any addition of noise or induced loss of competency, we observed that both the mean AIS and TE are decreasing during development and aging. For some organisms, we can see that TE can increase during aging, indicating episodes of regeneration in the sense that the morphology is impaired and the organisms try to recover. As we can observe it, the spatial entropy is slowly decreasing during aging, and the anatomy is slowly impaired, with the loss of organs such as eyes (see Figure 6).

Interestingly, we observe that in the cases where we increase noise, see Section 4.2.1, there is a threshold value for increasing the noise level after which a rapid decline in fitness occurs ‐ which we interpret as rapid aging/dying. Indeed, at the same moment, the SE is decreasing, showing a complete disappearance of the anatomy towards a quasi‐random distribution of the states. In this case, the AIS and TE increase around 600 steps, which is a bit past the half‐life mark. A high TE can be interpreted as a high entropy of actions, with the cells trying to act after the decline and disappearance of the anatomy.

However, the loss of cell competency does not impact the AIS and TE immediately as in the noise case. Instead, the effect on AIS and TE is delayed; we observe the onset of a lot of random states in the anatomy, with an increase in AIS and TE at the end of the organism's lifespan. And when both increases in noise and loss of cell competency are present, the increases in AIS and TE are higher than when noise alone is present. Maybe the loss of competency alters the capacity of the cells to understand the decline of the anatomy and their potentials to counteract it. On the other hand, noise clearly creates a lot of cellular actions during the organism's lifespan.

When the connectivity of the cells is altered, after 500 steps we can observe an increase in AIS and more variability in TE (decrease and increase during aging). With the closing of the gap junctions, the cells are not able to receive a steady flow of information; this increases the uncertainty about their actions. We can see with the SE that the cells can counteract the loss of connectivity up to about 500 steps, after which they experience a significant decrease in spatial entropy and a complete inversion of the states of the anatomy.

The case that is creating the highest variability in anatomical outcome is the one where we altered the ANN parameters. AIS decreases and then increases slightly over time, TE is close to zero after development, and SE shows three different types of aging: a loss of structure (organs), proliferation of a specific states in the tissue (cancer), and random activation of cell states.

We can conclude that if the states of the organisms do not change over time – if the anatomy reached a steady state – the AIS and TE are low. AIS is low because there is no new information to be gained from observing past states – future states are always the same and known, independent of the past. Essentially, the predictability is absolute, but since the system does not utilize past information to determine this (because the state is intrinsically static), the AIS has low values. This indicates that in a non‐changing system there is no information flow necessary between past and future states. The future state is always known (its the same as the current state), and thus the storage of information from past states is irrelevant for future predictions. However, when AIS increases, it is also associated with an increase in TE. It happens in the cases of catastrophic changes in the morphology, as related to changes in spatial entropy. Important morphological change implies an important information flow between cells, possibly representing the cellular attempts to recover from the high morphological changes. The absence of information flow in the default behavior can indicate that the system considers its goal as reached, i.e., since neither development nor morphostasis are among the goals of the organism at this point, we see a steady degradation of the anatomy, which we can equate to aging. SE measures the entropy of the grid states. When SE is very high it corresponds to a random distribution of the states over the tissue. This random distribution can be found mainly in the cases with competency loss alone, noise alone, or both noise and competency loss. SE reveals also a different kind of aging corresponding to a loss of structure, with the loss of specific organs or the proliferation of a specific states in the tissue, as in the loss of connectivity and ANN parameters cases, meaning that the different perturbations lead to different kind of morphological degradation.

### 4.4 Regeneration as the Cure for Aging

#### 4.4.1 Loss of Organs Does Not Imply the Loss of Information About the Organ

In biological systems, certain cells or tissues retain “memories” of past states, influencing future behaviors. For instance, epigenetic memory in cells allows them to “remember” previous environmental exposures or developmental cues. Even if the external trigger is removed, cells can maintain certain gene expression patterns or epigenetic marks.^[^ ^95^ ^]^ Similarly, tissues may retain information about past structural organization. For example, some amphibians can regenerate limbs or eyes after they are entirely removed.^[^ ^96^, ^97^, ^98^ ^]^ Planarian flatworms exhibit a kind of holographic memory where, upon being cut into many pieces, each fragment of the animal regenerates everything that is missing to create a perfect and complete copy.^[^ ^99^, ^100^ ^]^ This persistence of information about the systems target morphology, which serves as the setpoint for the process of anatomical homeostasis, can be implemented in a variety of biochemical, bioelectrical, and biomechanical information; thus it is important to study this important question.

**Figure 7:**
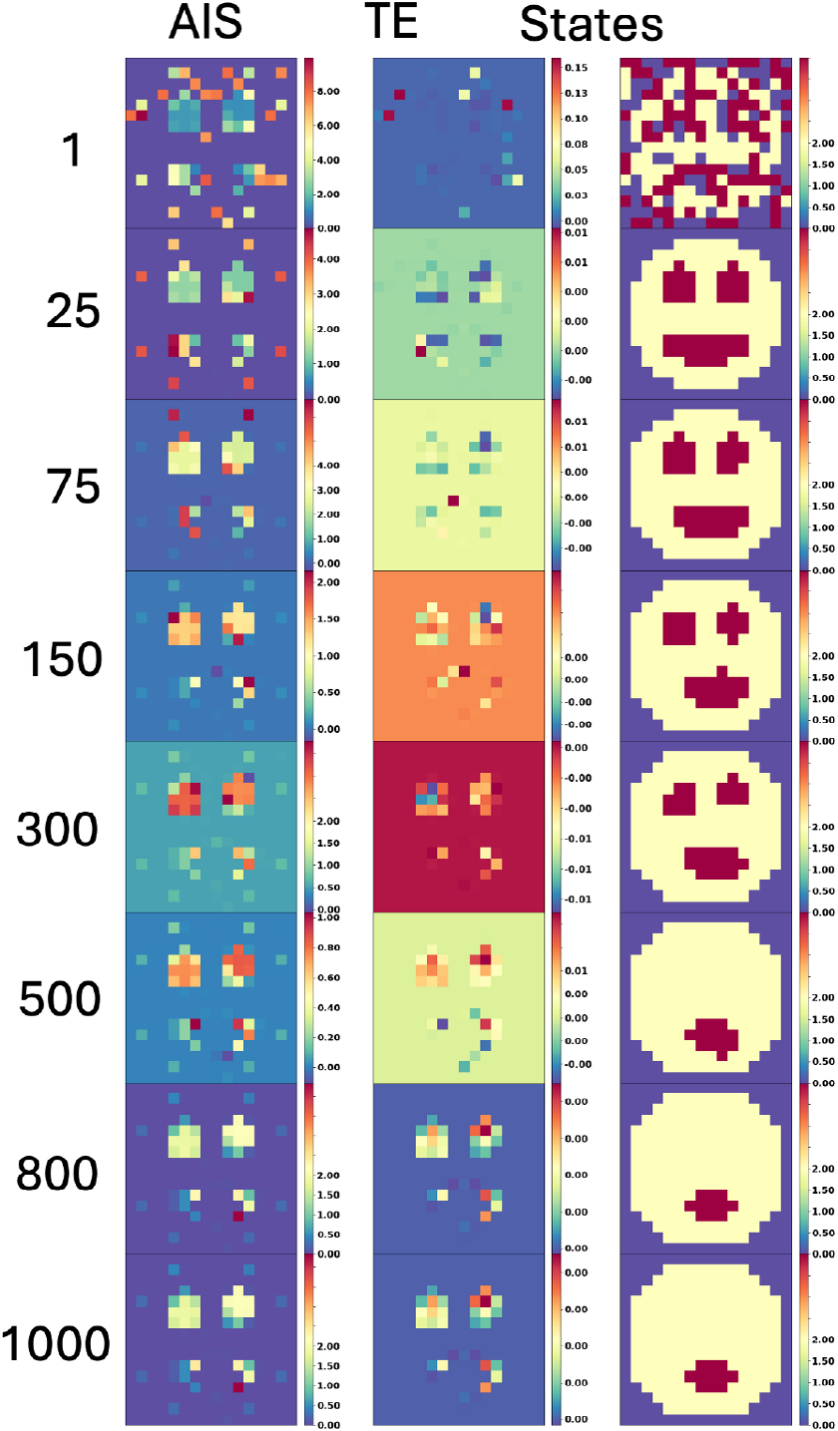
Loss of the organs but not of the information about the organ during normal aging. In order to study the information dynamics during aging and morphological decay, we applied AIS and TE during development and aging. Here is an example of a morphogenetic experiment where the organism is losing organs, here the eyes of the smiley face at step 500 in States column. But both AIS and TE are high at the spatial location of these organs (from 500 to 800 steps). This suggests memory retention, similarly to regenerative organisms that can regrow lost organs or limbs, keeping some memory of the lost body parts. This result will lead to the organ regeneration experiment (see **Figure** **8**).

In order to analyze the information dynamics during loss of organs, we computed the spatial AIS and TE (see **Figure** **7**). We observed that the loss of the eyes during aging does not imply the loss of the AIS at the location of the eye, as we can see at step 800 of Figure 7. The AIS is high and located at the position of the eyes in 100% of the cases. This suggests a memory retention in the system. For example, high AIS values at the “eye” locations in our system parallels the prepattern of the developing amphibian face consisting of regions of specialized cellular resting potential that determine the organs.^[^ ^101^ ^]^ Even after visible features are lost, underlying patterns (like bioelectric signals or AIS) can persist, reflecting the system's inherent memory of structure. AIS measures how much information about the past states is retained in a system over time. This retention can create a lingering effect, wherein regions that previously had distinct structures (like the eyes) continue to show high AIS values. Even if the explicit structure is no longer present (e.g., the “eyes” disappear in the smiley face), the underlying system dynamics may still carry information related to those past configurations. This residual AIS indicates that the system “remembers” these regions as important parts of its past structure, maintaining high information storage due to the prior role they played.

We conclude that the loss of organs does not imply the loss of information about the organ (see example Figure 7). Following this result we decided to explore the implications for rejuvenation by inducing the regeneration of the organs in the system in the next section.

#### 4.4.2 Implication for Rejuvenation: A Simulated Experiment of Organ Restoration

The main reason for the long‐term fitness degradation in our experiments ‐ as discussed in Section 4.1 (i.e., even without artificially affected cellular competencies), is the loss of specific internal organs, such as the eyes or the mouth. Thus, we here aim to correspondingly counteract the loss of detrimentally affected organs by replacing either the entire organ or specifically reconfiguring the affected cells of the NCA with “regenerative” state information (also erasing their internal working memory by resetting their RNN state information; see Appendix A in^[^ ^46^ ^]^ for details).

**Figure 8:**
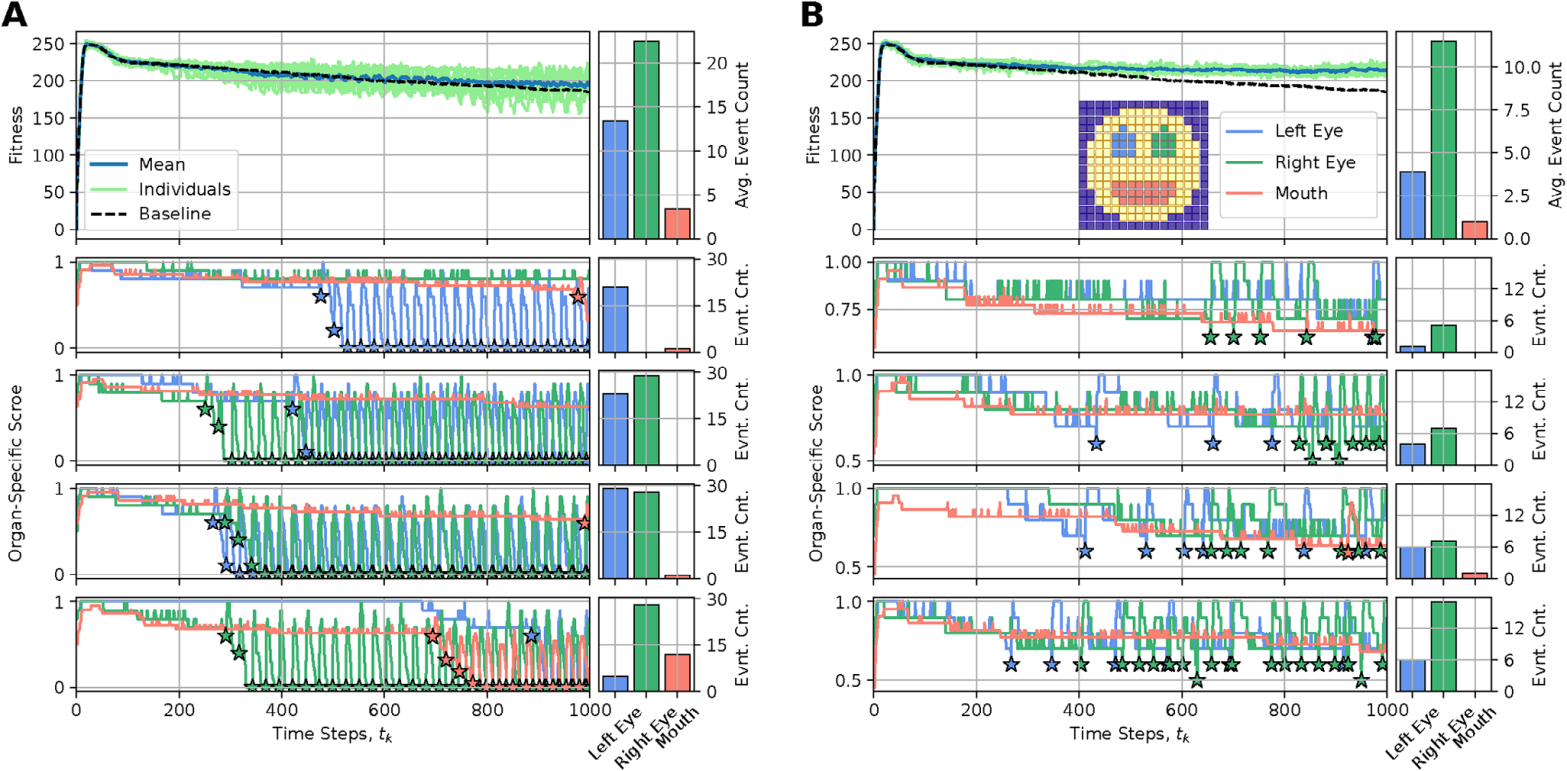
Organ restoration experiments. (A) Replacing the entire affected organs, i.e., the left eye (blue color code), the right eye (green colored), and the mouth (red colored) with their corresponding initial states if the respective organ‐specific score is lower than the threshold score of *r*_th_ = 0.6. (B): same as (A) but reconfiguring only the wrongly expressed cells, not the entire organ, of an affected organ. In both, (A, B), the top‐left panel presents the mean fitness score (blue) of *ten* statistically independent individual simulations of the respective recovery experiments (green), which we contrast with the baseline case without recovery depicted in Figure 4. The four bottom‐left panels in (A) and (B) respectively present the organ‐specific scores (color‐coded) of four selected individual lifespan simulations from the top panels; colored stars indicate rejuvenation events. The bar plots indicate the total number of organ‐specific rejuvenation events for all trajectories (top right panel) and the selected individual trajectories (bottom right panels) again respectively for (A) and (B). The cartoon inset in the top panel of (B) indicates the respective loci in the NCA used to evaluate the respective organ‐specific fitness score (i.e., the blue, green, and red organ masks). While the strategy in (A) of replacing detrimentally affected organs entirely with their respective primordial cells does not seem effective (many repetitive interventions are required as a form of symptom control), for the majority of individual simulations in (B), typically only very few intervention events of resetting the affected cells of a corrupted organ to their primordial state suffice to restore sustainable system‐level performance.

To achieve this, we define an organ‐specific score function, $r_{o}\left(t_{k}\right)=N_{\hat{g}}\left(t_{k}\right)/{\hat{N}}_{o}$, that quantifies for every time‐step *t*_*k*_ the fraction of correctly expressed target cells $N_{\hat{g}}\left(t_{k}\right)$ out of the total number of target cells ${\hat{N}}_{o}$ of a specific organ, o∈{LeftEye,RightEye,Mouth} (c.f., blue, green, and red cells in the snapshot of the 16 × 16 smiley‐face pattern NCA in Figure 8, respectively). Whenever an organ's score falls below a predefined threshold value of *r*_o_(*t*_*k*_) < *r*_th_, we perform an intervention to the affected organ, aiming at restoring its morphology locally. To interfere as little as possible with the NCA's inherent developmental program, we propose utilizing the initial cell states as the regenerative state information of affected cells, $c_{i}^{*}\left(t_{k}\right)\to c_{i}\left(0\right)$ (c.f., Section 2.1). This corresponds to reconfiguring the affected cells to their initial (primordial) state at the onset of the developmental program of the NCA.

#### 4.4.3 Less is More: Organ Restoration Induction by Injecting the Regenerative Information only to Incorrectly‐Positioned Cells

We thus deploy the NCA solution discussed in Section 4.1 (c.f., Figure 4) for an extended life‐span of *t*_*A*_ = 1000 time steps and apply the here discussed rejuvenation interventions whenever an organ's score *r*_o_(*t*_*k*_) falls below a threshold value of *r*_th_ = 0.6. In Figure 8A,B, we respectively present the results of this procedure by either (A) resetting all cells of an affected organ with their initial configuration or (B) only resetting the particular cells of an affected organ that express different cell states than their target types. We present for both (A) and (B) the long‐term fitness scores of 10 statistically independent simulations, contrast their mean behavior with the baseline case without interventions (see Figure 4), and visualize the organ‐specific scores and the corresponding intervention events for selected individuals.

We see that method (A) is not very sustainable: Although we generally measure significant short‐term improvements in a respective organs' scores after such intervention events, the intervened organs dissolve again quickly so that after ≈25 time steps (which we set as the lower limit of two consecutive interventions, allowing for unperturbed development during this period) the organ needs to be reset again, and again. This periodic need for interventions ‐ also reflected in correspondingly oscillating fitness scores of the entire organism ‐ seems fundamental in our system and is not significantly affected by the delay between two interventions. The resulting fitness scores of the intervened individuals are only slightly improved compared to the baseline case.

Strikingly, if only the wrongly expressed cells of an affected organ are reconfigured to their initial state, as in case (B), very few of such targeted interventions (on the order of ≈5 − 15, c.f., bar plots in Figure 8B) are sufficient to sustainably restore degrading organs and allow the corresponding virtual organism to maintain its morphological integrity over an extended lifespan. The corresponding long‐term fitness scores of the rejuvenated individuals show significant improvement compared to the baseline case and can even be stabilized into a steady state of a constant value of, here, *r*_A_ ≈ 200; notably, the difference to the maximum fitness score of *r*_max_ = 256 right after the developmental stage at *t*_D_ = 35 arises due to the accumulative stagnation loss term *r*_S_ in Equation (5).

#### 4.4.4 Boundaries Matter: Organ Restoration is More Efficient with the Injection of a Differential Pattern Including the Organ Cell States and Neighboring Cell States

**Figure 9:**
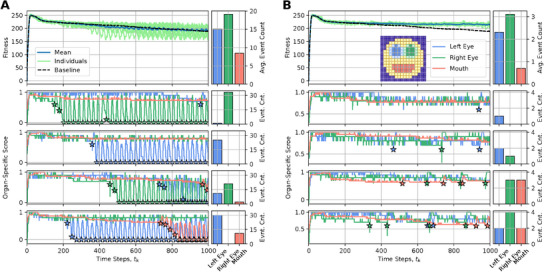
Organ and surrounding tissue restoration experiments. Similar to Figure 8 but here considering not only the respective organ cells but also the cells at the boundary to adjacent tissue in detecting affected organs and corresponding interventions (c.f., the eye sockets colored in light‐blue and light‐green for the left and right eyes, respectively). Notably, while resetting the entire affected organ and its surrounding tissue (A) shows similar performance to resetting only the organ (see Figure 8A), considerably fewer interventions are required in (B) to achieve the same effects as in Figure 8B when including the boundaries to reconfigure wrongly expressed cells of the respective affected organs.

Although the above approach is already promising, we further investigate how including the cells at the boundary to adjacent tissue surrounding the respective organs affects regeneration in our procedure. Thus, we additionally consider the respective boundary cells for the left and right eyes in the evaluation of the respective organs' scores, but we intentionally omit the boundaries of the mouth (c.f., emphasized color‐coded cells in **Figure** **9**, similar to Figure 8); notably, the mouth is much more stable than the eyes, and its cells already occupy a substantial fraction of the face.

Deploying the same experiments as above in Section 4.4.3, we see in Figure 9 that (A) interventions reconfiguring all the cells of affected organs (now also including their respective surrounding tissue) show similar performance to the corresponding case shown in Figure 9A. However, the more targeted cell‐specific interventions depicted in Figure 9B – manipulating not only the wrongly expressed cells of an affected organ, but also the wrongly expressed cells of their respective boundaries – greatly improve the performance of restoration interventions as compared to the cases shown in Figures 8, 9 and Figure 9B. Here, an average number of as few as 5 − 6 interventions (in some cases even 1 or 2) suffice to stabilize the morphology of the here investigated 16 × 16 smiley‐face pattern organism in long lifespan experiments.

We conclude that boundaries (and more generally, relations) between organs matter in our regeneration experiments: including surrounding tissue in detecting affected organs and in subsequent regeneration experiments that specifically target the wrongly expressed cells of the affected organs can be an efficient approach for regeneration and rejuvenation applications in our system.

A different but likewise promising approach is replacing the affected organs or cells directly with their target states (rather than their respective initial states as here). However, while “transplanting” entire organs can be effective, there is little quantitative difference in rejuvenation performance between replacing cells with their target states and reconfiguring cells into their initial states. From a biological perspective, the latter approach of resetting cells to their primordial states strikes us as conceptually and technologically more feasible, as the number of specialized target states might greatly exceed the number of necessary primordial initial states: resetting cells to their initial cell state, or at least to a particular primordial cell state suitable for starting a specific morphogenetic task, could serve as a general fallback mechanism to induce regeneration of the respective (here virtual) organism by restarting the developmental program locally.

At this point, we would like to stress that the developmental program of our NCA has not been optimized either for such long lifespans or for the here‐proposed intervention experiments. The latter can thus be seen as minimally invasive ad‐hoc cellular reconfiguration interventions, i.e. a dynamic and targeted “hacking” of the organism's developmental plan by an external agent,^[^ ^23^ ^]^ with the external agent in this case being the researchers (us).

Overall, we conclude that such a procedure of targeted cellular interventions is a reliable approach for rejuvenation in our system.

## 5 Discussion and Conclusion

In this paper, we aim to address a significant gap in the aging literature which, to the best of our knowledge, so far lacks computational models of aging considering cellular information processing and multi‐scale competency. We seek to address this knowledge gap by developing an evolutionary framework integrating the multi‐scale competency architecture of multicellular tissue.

Specifically, we here utilize numerical tools from Artificial Life,^[^ ^66^ ^]^ so‐called Neural Cellular Automata (NCAs)^[^ ^44^, ^46^, ^72^ ^]^ which we trained with an evolutionary algorithm to perform self‐orchestrated targeted pattern formation, i.e., morphogenesis of a 16 × 16 smiley‐face pattern. The localized unicellular decision‐making centers in our NCA implementation are modeled by Artificial Neural Networks (ANNs), which perceive the numerical states of their direct cellular neighbors on the grid of the NCA to regulate their own cell state individually. In that way, NCAs substitute homeostatic and morphogenetic pathways of “real” multicellular biological tissue, with the NCA‐specific ANNs and numerical states respectively corresponding to cell‐internal decision‐making machinery (such as gene regulatory networks) and cell‐type expressions.

Analogously to their biological counterparts, the NCA's cells form their decisions following a global agenda, allowing the collective of cells to assume a predefined target pattern over a predefined developmental time, thus simulating the process of embryogenesis. The evolutionary algorithm selects mature phenotypes directly after the predefined developmental stage (i.e., fully grown tissue), specifically quantifying how well the cells of the corresponding NCAs can grow the facial features and organs of the 16 × 16 smiley‐face pattern in a fully self‐orchestrated and decentralized way. Notably, the NCA's cells do not in any way explicitly perceive time or their own age (in terms of simulation time steps), but their dynamics are fully governed by regulating and exchanging noisy state information on the NCA's grid. Yet, when deploying a successfully trained NCA for much longer times than necessary for development (at which stage evolutionary selection would have occurred), the tissue of the NCA expresses signs of aging, such as successively blurring boundaries between organs, or entirely losing one or more organs. We emphasize that this behavior is emergent and happens without any further intervention.

We propose that, even in the absence of accumulated cellular or genetic damage, a biological system left without any goal in the morphospace will start degrading anatomically. Using an in silico model of homeostatic morphogenesis with a multi‐scale competency architecture and information dynamics analysis, which provide a novel perspective on aging dynamics with significant implications for longevity research and regenerative medicine, we found the following: 1) Absence of long‐term morphostasis goal: aging emerges naturally after development due to the lack of an evolved regenerative goal, rather than being caused by specific detrimental properties of developmental programs (e.g., antagonistic pleiotropy or hyperfunction); 2) Acceleration factors versus root cause: cellular misdifferentiation, reduced competency, communication failures, and genetic damage all accelerate aging but are not its primary cause; 3) Information dynamics in aging: aging correlates with increased active information storage and transfer entropy, while spatial entropy measures distinguish two dynamics, i) the loss of structure and ii) morphological noise accumulation; 4) Dormant regenerative potential: despite organ loss, spatial information persists in our cybernetic tissue, indicating a memory of lost structures, which can be reactivated for organ restoration through targeted regenerative information; and 5) Optimized regeneration strategies: restoration is most efficient when regenerative information includes differential patterns of affected cells and their neighboring tissue, highlighting strategies and protocols for rejuvenation.

While the cells in our system learn to perform morphogenesis, i.e., how to grow an adult morphology successfully, we still observe aging effects in long‐term simulations even without artificially perturbing the NCAs parameters. These results suggest that development and morphostasis, while close in terms of morphological trajectories and tasks, still significantly diverge. To counteract aging, it is therefore necessary to (re)activate a developmental/regenerative goal. We explicitly demonstrated this by providing our cybernetic tissue with the necessary information for regeneration, thereby inducing rejuvenation through self‐organization at the anatomical level. Our results suggest a roadmap for rejuvenation therapies, particularly that reversing aging may imply providing a new goal to the tissue, e.g., regeneration. In other words, this suggests that activating regeneration may stop or reverse aging. As currently debated, regenerative species such as salamanders or planaria might not age at all. While already known for planaria, this is the subject of current research on salamanders. The latter not only lack senescent phenotypes, but it is indeed impossible to define an epigenetic clock for a specimen older than four years.^[^ ^102^ ^]^ One extension to the present work will therefore involve comparing an aging system that has only been trained on morphogenesis tasks to a system that has learned to regenerate and then actively maintain morphostasis after development. Understanding their differences in terms of information dynamics will allow us to form a thorough understanding of the homeostatic loops that allow differently expressed cells in a self‐assembling tissue to maintain their integrity against adversarial attacks or thermodynamic noise.

This work also emphasized how to optimize regenerative strategies. Our results show the importance of the information at the level of the borders of integrated tissue or organs for regeneration. This importance can be found in the field of bioelectricity. Indeed, research indicates that mutations in critical neurogenesis genes, such as Notch, can lead to developmental defects.^[^ ^103^ ^]^ These organ defects can be cured through the overexpression or activation of native HCN2 (hyperpolarization‐activated cyclic nucleotide‐gated) channels.^[^ ^104^, ^105^, ^106^ ^]^ As voltage‐sensitive ion channels, HCN2 channels respond to their cellular environment by enhancing hyperpolarization in cells that are slightly polarized, while remaining inactive in depolarized cells. This mechanism functions analogously to “contrast enhancers,” which sharpen weak differences in membrane potential (V_mem_) across compartment boundaries, much as a sharpening filter brings clarity to a blurred image. In other words, HCN2 enhances and sharpens the borders in the bioelectrical pattern.

Moreover, our results suggest that a multicellular collective is capable of maintaining the memory about fully degraded features of an organism, as explicitly demonstrated here via organ regeneration experiment and information dynamics analysis. This suggests that this information is dormant and can be “retrieved” by injecting the appropriate signals that trigger regeneration in our system. This finding opens up exciting possibilities for future research, particularly in the realm of regenerative medicine and bioengineering. By understanding how cells store and retrieve hierarchical anatomical information, we could potentially develop new methods to promote regeneration in damaged tissues or organs. Additionally, exploring the mechanisms by which cells communicate and coordinate to maintain this memory could lead to breakthroughs in preventing age‐related degeneration. For example, targeting specific bioelectrical patterns or enhancing the function of key ion channels like HCN2 could help maintain tissue integrity and function over time. Furthermore, the here‐discussed principles targeting the software of life could be applied to synthetic biology and rejuvenation, where designing artificial systems with similar regenerative capabilities^[^ ^107^, ^108^ ^]^ as well as new aging treatments might become feasible.^[^ ^31^, ^39^ ^]^

While our current framework is capable of self‐assembling target patterns of heterogeneously distributed cell types, the ANN‐based decision‐making centers of our NCA's cells are implemented homogeneously: all cells of an NCA typically share the same ANN structure and parameters, and thus the same basic functionality. Yet, the cells might be configured differently and thus effectively behave heterogeneously due to cell‐specific state expressions and input relations, in short, due to their morphogenetic history. In that way, these artificial cells' functionality mirrors gene regulatory networks. Thus, further in silico studies could investigate minimally complex high‐level interventions necessary to induce robust regenerative effects in affected tissue simply by reconfiguring the cellular collective locally. Furthermore, allowing cell‐specific functional mutations during the lifetime of such virtual organisms might be an intriguing way to study morphogenetic and aging‐related implications of heterogeneity.

Our results validate aspects of both damage‐based and programmatic theories of aging^[^ ^1^, ^4^, ^5^ ^]^ but in very different ways. On the one hand, accelerated morphological decline (or an increased rate of aging) in our system mainly originates from: increasing cellular misdiffentiation, decreasing cellular competency, defective cell–cell communication, and accumulation of genetic damage. While these four characteristics align with damage‐based theories of aging,^[^ ^6^ ^]^ they are not really causal, as our system also undergoes aging in their absence. Therefore, our model tends toward programmatic theories of aging, antagonistic pleiotropy or hyperfunction,^[^ ^16^, ^17^ ^]^ or the theories of the software design flaw,^[^ ^5^, ^31^ ^]^ in which the causes of aging have to be found in development. Similarly, but not analogously, we here relate aging to the absence of a developmental/regenerative or morphostatic goal, which nonetheless may imply an abnormal continuation of specific developmental pathways that are not beneficial for anatomical homeostasis. However, our results suggest that developmental programs (learned during evolution) can also qualify as regenerative pathways when “reactivated” in aging individuals, thus representing a potential protocol for a cure for aging (similarly to^[^ ^109^ ^]^). In a sense, regeneration is learned for free and is merely dormant after development. This is an important result and indicates that we should focus our research efforts for curing aging on the reactivation of the regenerative capabilities. Additionally, our results suggest an experimental research program for regenerative medicine wherein we create an embryo‐like environment allowing the tissue to regenerate. In turn, when interpreting the NCAs' numerical states as bioelectric degrees of freedom, instead of transcriptomic cell‐state expressions, a promising approach toward innovative no‐aging or rejuvenation treatments might be to focus on high‐level control layers such as bioelectricity that encode collective anatomical goals.^[^ ^31^, ^107^ ^]^

The observation that spatial information about lost organs persists in the cybernetic tissue suggests a form of morphological memory that could be harnessed for regeneration. This resonates with the findings of Deng and Terunuma (2024), who review the potential of adoptive natural killer (NK) cell therapy in clearing senescent cells (SNCs) and alleviating senescence‐associated secretory phenotypes (SASPs) to promote longevity.^[^ ^110^ ^]^ In both frameworks, aging involves the obstruction of inherent restorative capabilities – morphological setpoints in our cybernetic tissue or latent regenerative programs in biological systems, which can be reactivated: our model does so by leveraging retained information for anatomical restoration, while NK therapy removes dysfunctional cells to allow the body's natural repair processes to re‐emerge, ultimately promoting tissue homeostasis and longevity.

Our study showed fundamental computational principles in multicellular aging systems, bridging developmental biology and aging research. We explicitly demonstrate that developmental pathways, initially used for morphogenesis, may be dormant and can be reactivated for regeneration and tissue maintenance later in life. Moreover, we observe that cells collectively retain and manipulate morphological information, indicating that targeted interventions could potentially rejuvenate or maintain tissue integrity as organisms age.

While our model offers conceptual insights into aging as a loss of goal‐directedness in morphospace, several limitations must be highlighted. The individual cells in our NCA framework are highly simplified, lacking the molecular, genetic, and signaling complexities of real biological cells, although the present NCA approach is flexible enough to provide a simulation component to a wide variety of known biological mechanisms that might be found to be involved in future work. The simulated multicellular systems are small‐scale, as are organoids currently used to screen various interventions, rather than capturing the vast complexity of full organs or entire organisms like humans. Additionally, we did not include genetic control after development and selection shadow. Although the simulations illustrate the plausibility of developmental systems evolving for maturation but not long‐term maintenance, this does not empirically prove that such mechanisms drive biological aging. These constraints highlight the need for future empirical validation and more biologically detailed models to bridge the gap between model predictions and real‐world applicability. This experimental work is however ongoing; indeed, senescent cells show a particular voltage membrane depolarization^[^ ^111^ ^]^ and their accumulation may corrupt the bioloelectrical pattern in charge of the anatomical goal.^[^ ^39^ ^]^ It also remains critical to understand and model the dynamics of pattern memory in organisms that do not age (e.g., asexual planaria), which is an on‐going research direction in our lab.

Our loss of goal‐directedness paradigm of aging may also lead to testable predictions with existing data and raises fundamental questions about the relationship between growth patterns, goal‐directedness, and aging across biological systems. Our model predicts that indeterminate growth patterns (or regeneration) should correlate with minimal aging phenotypes, as persistent morphogenetic goals would prevent the loss of anatomical homeostasis goal that characterizes aging. This aligns with observations of negligible senescence and extreme longevity in species like lobsters, certain sharks, planaria for continuous regeneration, and some trees and fungi that maintain continuous growth throughout their lifespans.^[^ ^112^, ^113^, ^114^, ^115^, ^116^ ^]^ However, this raises the paradoxical question of why natural selection hasn't universally favored slow, continuous growth as an anti‐aging strategy. The answer may lie in energetic trade‐offs and ecological constraints: maintaining active morphogenetic programs requires substantial metabolic investment that may compromise reproductive success or survival under resource limitation.^[^ ^117^, ^118^ ^]^ Our model also predicts that organisms with indeterminate growth should exhibit reduced cancer incidence, as continuous morphogenetic goal‐directedness would maintain cellular coordination toward system‐level objectives rather than allowing cells to pursue lower cellular goals like cancer,^[^ ^93^ ^]^ something we can also observe in the species mentioned above showing continuous growth.^[^ ^115^, ^119^, ^120^ ^]^ On the other hand, evolving ecosystems can be seen as learning systems.^[^ ^121^ ^]^ Populations of ever‐expanding, undying individuals with limited variability would dominate the gene pool, limiting generational turnover.^[^ ^122^ ^]^ In contrast, organisms with finite lifespans ensure continual renewal, effectively enhancing adaptability at the system level,^[^ ^123^ ^]^ positioning aging as a potential mechanism for promoting evolvability at the population level.^[^ ^124^, ^125^, ^126^ ^]^

Overall, we focused on the loss of goal‐directedness in morphospace as the cause of the degradation within the system described in this article. However, it is also conceivable that loss of goal‐directedness could cause degradation not only within morphospace, but also at other levels within both biological and artificial systems. For example, it may be possible to counteract psychological decline during aging by providing new life goals to the organism, which is in line with resistance to cognitive decline that has been found in elderly having high engagement in social and/or physical activities.^[^ ^127^, ^128^, ^129^ ^]^ At another level, developing new goals can also be accomplished by providing new patterns within a body (as occurs in metamorphosis^[^ ^130^, ^131^ ^]^), or via communication of signals between bodies, as recently observed in cross‐embryo morphogenetic error correction effects.^[^ ^132^ ^]^ In terms of learning theory, we could ask: what is the objective of a goal‐directed system once it reached its goal? Our results may point towards the idea that a goal‐directed system that has achieved its goal becomes an aging system in a broad sense: a system slowly deviating from its goal. For a morphogenetic system, this might be reflected by a slowly degrading anatomy, a slight shift in morphospace^[^ ^26^ ^]^ ultimately leading to death: an anatomical configuration incompatible with life. For AI systems, a decline in performance, similarly to what has been seen with ChatGPT or other LLMs,^[^ ^133^ ^]^ could be interpreted as aging or developmental defects after the target morphology has been reached.^[^ ^44^ ^]^ Our results suggest that reaching a goal by generative means is completely different from maintaining it. Thus, to engineer robust and autonomous AI and intelligent robotic systems or synthetic morphology we should integrate the learning of these two different tasks.

Exploring these principles further could lead to significant advancements in delaying or reversing age‐related decline or diseases. Indeed, the loss of goal‐directedness at the morphogenetic level allows the cells to pursue their own goals; cancer (an aging‐related disease) may be seen as one such new cellular goal.^[^ ^93^ ^]^ Integrating computational models with experimental biology might pave the way to uncover new strategies to enhance regeneration through manipulating cellular behavior, potentially extending the healthy lifespan of tissues.

Future research will focus on applying these computational insights to real‐world biological systems, aiming to develop therapies that modulate aging processes and improve recovery from injuries, thus optimizing the health span of multicellular organisms. These findings not only deepen our understanding of the aging process but also highlight the potential of using computational approaches to predict and control the dynamics of cellular systems in aging. The insights gained from this study open new avenues for research in aging and regenerative medicine, paving the way for novel interventions that could one day transform our approach to aging and chronic disease treatment.

## Conflict of Interest

The Levin lab has a sponsored research agreement with Astonishing Labs, a company seeking to impact the longevity and biomedicine of aging field.

## Data Availability

The data that support the findings of this study are available from the corresponding author upon reasonable request.
